# Metabolic profiling and antibacterial activity of tree wood extracts obtained under variable extraction conditions

**DOI:** 10.1007/s11306-024-02215-x

**Published:** 2024-12-27

**Authors:** Diana Vinchira-Villarraga, Sabrine Dhaouadi, Vanja Milenkovic, Jiaqi Wei, Emily R. Grace, Katherine G. Hinton, Amy J. Webster, Andrea Vadillo-Dieguez, Sophie E. Powell, Naina Korotania, Leonardo Castellanos, Freddy A. Ramos, Richard J. Harrison, Mojgan Rabiey, Robert W. Jackson

**Affiliations:** 1 School of Biosciences and the Birmingham Institute of Forest Research, University of Birmingham, Birmingham, B15 2TT UK; 2 Facultad de Ciencias, Departamento de Química, Universidad Nacional de Colombia - Sede Bogotá, Carrera 30# 45-03, Bogotá, D.C 111321 Colombia; 3 Plant Sciences Group, Wageningen University & Research, Wageningen, 6700AA The Netherlands; 4 School of Life Sciences, Gibbet Hill Campus, University of Warwick, Coventry, CV4 7AL UK

**Keywords:** Extraction, Mass spectrometry, Tree-metabolomics, Wood, Chemical diversity

## Abstract

**Introduction:**

Tree bacterial diseases are a threat in forestry due to their increasing incidence and severity. Understanding tree defence mechanisms requires evaluating metabolic changes arising during infection. Metabolite extraction affects the chemical diversity of the samples and, therefore, the biological relevance of the data. Metabolite extraction has been standardized for several biological models. However, little information is available regarding how it influences wood extract’s chemical diversity.

**Objectives:**

This study aimed to develop a methodological approach to obtain extracts from different tree species with the highest reproducibility and chemical diversity possible, to ensure proper coverage of the trees’ metabolome.

**Methods:**

A full factorial design was used to evaluate the effect of solvent type, extraction temperature and number of extraction cycles on the metabolic profile, chemical diversity and antibacterial activity of four tree species.

**Results:**

Solvent, temperature and their interaction significantly affected the extracts’ chemical diversity, while the number of extraction cycles positively correlated with yield and antibacterial activity. Although 60% of the features were recovered in all the tested conditions, differences in the presence and abundance of specific chemical classes per tree were observed, including organooxygen compounds, prenol lipids, carboxylic acids, and flavonoids.

**Conclusions:**

Each tree species has a unique metabolic profile, which means that no single protocol is universally effective. Extraction at 50 °C for three cycles using 80% methanol or chloroform/methanol/water showed the best results and is suggested for studying wood metabolome. These observations highlight the need to tailor extraction protocols to each tree species to ensure comprehensive metabolome coverage for metabolic profiling.

**Supplementary Information:**

The online version contains supplementary material available at 10.1007/s11306-024-02215-x.

## Introduction

Trees play a vital role in supporting biodiversity in forests (Asbeck et al., 2021), serving as carbon sink (Mitchard, 2018), and offering a source of food, materials and bioactive compounds (Abedini et al., 2020; Elansary et al., 2019; Hayat et al., 2022; McEwan et al., 2020; Newman & Cragg, 2020; Szwajkowska-Michałek et al., 2020; Willig et al., 2022). As with any other organism, trees are susceptible to bacterial diseases that reduce their lifespan and productivity. Among these, diseases affecting the wood, such as oak bleeding canker associated with acute oak decline (AOD), bacterial canker of cherry and ash, and bleeding canker of horse chestnut, have become increasingly prevalent in the UK, causing severe damage in orchards, nurseries and forests (Denman et al., 2014, 2016, 2018; Green et al., 2009; Hulin et al., 2018, 2020; James et al., 2020; Janse, 1981; Moreno-Perez et al., 2020; Webber et al., 2008). Despite the importance of these tree-pathogen interactions, the understanding of the disease development and tree defence mechanisms remains limited.

Untargeted metabolomics of plant-pathogen interaction significantly helps the understanding of disease development and has helped in the generation of effective control strategies, such as the selection of resistant plant genotypes (Castro-Moretti et al., 2020; Fiehn, 2002; Yang et al., 2022). For example, untargeted metabolomics was used in selecting resistant phenotypes of beans against *Pseudomonas savastanoi* pv. *phaseolicola*. In this study, specific metabolites, such as salicylic acid, were observed to accumulate significantly in resistant plants at the site of infection, revealing metabolic pathways associated with effective defence mechanisms against pathogens (Cooper et al., 2022)​. Similarly, Nemesio-Gorriz et al. (2020) and Sidda et al. (2020), found that the tolerance or susceptibility of ash trees towards the ash dieback (AD) fungus, *Hymenoscyphus fraxineus*, was associated with the abundance of diverse metabolites including secoiridoids (Nemesio-Gorriz et al., 2020; Sidda et al., 2020), flavonoids, lignans, and coumarins (Nemesio-Gorriz et al., 2020). This allowed the characterisation of AD-tolerant and AD-susceptible chemotypes in ash, and enhancing understanding about the defence mechanisms employed by this tree against plant pathogens.

Despite its usefulness, plant metabolomics can be highly complex due to the wide chemical diversity of plants, metabolites variable concentrations, and low metabolite annotation rates (Alseekh & Fernie, 2018; Lee et al., 2020; Li & Gaquerel, 2021; Perez De Souza et al., 2021; Rai et al., 2017). To effectively approach these complex systems, it is essential to select the analytical platform and experimental design properly. High-resolution tandem mass spectrometry (MS^n^) is currently a widespread platform used for plant untargeted metabolomics due to its high resolution, sensitivity, and throughput (Rodrigues et al., 2021; Zhou et al., 2012). This platform provides structural information that, through different bioinformatic tools, facilitates the identification and classification of metabolites, enabling the annotation of unknown compounds (Alseekh & Fernie, 2018; Dreher, 2014; Dührkop et al., 2019; Horai et al., 2010; Ludwig et al., 2020; Nothias et al., 2020; Perez De Souza et al., 2020; Schmid et al., 2021; Smith et al., 2005).

To ensure the quality of the data acquired through MS and their biological relevance, an appropriate extraction system must be selected (Alseekh et al., 2021; Alseekh & Fernie, 2018). Typically, this decision relies on literature reports, with few standard protocols being available for model plants (Kim et al., 2010). In the case of trees, most data focus on studies related to leaves or fruits, with no consensus on which extraction system is most suitable (Rodrigues et al., 2021). Therefore, it is essential to evaluate different extraction protocols to determine the most suitable approach for studying tree woody tissues. This includes assessing various extraction factors, including type of solvent, temperature, and number of extraction cycles, that are known for affecting the coverage of metabolites obtained from the samples (Bijttebier et al., 2016; Canelas et al., 2009; Martin et al., 2014; Sostare et al., 2018; Zhou et al., 2012).

In this context, using published protocols to guide our study, eighteen methods were tested to determine the most effective extraction protocol for obtaining chemically diverse extracts from the woody tissues of four tree species: *Fraxinus excelsior* (ash), *Quercus* spp. (oak), *Aesculus hippocastanum* (horse chestnut), and *Prunus avium* (cherry). Among these methods, three solvents were used: 10% methanol, 80% methanol, and a variation of the Bligh and Dyer extraction solvent (chloroform/methanol/water, CMW) (Bligh & Dyer, 1959). Additionally, three extraction temperatures (4 °C, 20 °C, and 50 °C) and two numbers of extraction cycles (one and three) were considered. Each protocol’s extraction yield, chemical diversity, and antibacterial activity were evaluated and compared. Furthermore, the effects of each extraction factor on the chemical diversity and metabolic profile of the samples were examined. Finally, a comparison of the trees’ metabolic profile and activity was performed, allowing us to establish a baseline of the expected chemical space of each tree for future research.

## Methods

### Sample collection

Woody tissues (2 × 2 × 3 cm HeighxWidthxLength) were collected from the bottom branches of five asymptomatic ash, cherry, horse chestnut and oak trees from the Edgbaston campus at the University of Birmingham (Table S1) in June 2022. Before sampling, the outer bark was removed, and the inner bark and cambium were collected from three different branches per tree and flash-frozen at -80 °C. Afterwards, the samples were freeze-dried (LyoDry Benchtop, Mechatech Systems Ltd, UK), ground to fine dust in liquid nitrogen, and combined to create a pooled sample per tree species.

### Experimental design

A full-factorial design with mixed levels (1^2^2^3^) was used with variations in solvent (10% methanol, 80% methanol, CMW v/v 1/2.5/0.5), temperature (4 °C, 20 °C, 50 °C), and number of extraction cycles (one or three extractions). A total of 18 combinations (protocols, P1-P18) were evaluated in triplicate (Table S2). The extract was obtained by adding 500 µl of solvent to 100 mg of sample. The sample was mixed by vortex, ultrasonicated (20 min, 37 kHz, 80 W, Fisherbrand™ S-Series Heated Ultrasonic Bath FB-15051) on ice bath (4 °C), or at 20–50 °C, and centrifuged (10 min,13.000 rpm.). The obtained supernatant was dried in a Speedvac (45 °C, Eppendorf Concentrator plus™, Eppendorf AG, Germany), weighed, dissolved in 50% acetonitrile ([10 mg ml^− 1^], ACN) and stored at -20 °C. This procedure was repeated three times for the 3-cycle protocols, collecting the supernatant into a single tube. The extraction yield was defined as the amount of dry extract (mg) per ml recovered from each extraction protocol.

### HPLC-MS/MS analysis

10 µl of each extract was injected into a Dionex UltiMate 3000 chromatographic system coupled to a Q-Exactive mass spectrometer (Thermo Fisher Scientific, Germany), using a C18 column (Hypersil GOLD, 100 × 2.1 mm, particle size: 1.9 μm, Phenomenex, USA). The mobile phase comprised 95% H_2_O (Fisher Chemical™) + 0.1% formic acid (FA, Fisher Chemical™) as solvent A, and 95% ACN + 0.1% FA as solvent B. The flow rate was set to 250 µl/min. The chromatographic program was set to 0% B from 0 to 1.5 min, 0–99% B between 1.5 and 12.5 min, 99% B from 12.5 to 14 min, 0% B from 14 to 15 min and 0% B for 2 min. Extracts from each tree species were injected separately. For each dataset, a pooled sample (QC) was obtained by combining 10 µl of each sample. 50% ACN was used as blank control. Samples were injected randomly, including one QC and blank for every eighteen samples.

Data-dependent acquisition of MS/MS spectra was done in positive mode. Electrospray ionization (ESI) parameters were set as follows: 40 arbitrary units (AU) sheath gas flow, 10 AU auxiliary gas flow, 3 AU sweep gas flow, 100 °C auxiliary gas temperature, 3.9 kV spray voltage, 275 °C inlet capillary temperature and 50 V S-lens level. MS scan range was set to 100–1500 *m/z* with a resolution of 140,000. The maximum ion injection time was 100 ms with an automatic gain control (AGC) target of 1E6. Up to five MS/MS spectra per duty cycle were acquired with a resolution of 17,500. The maximum ion injection time for MS/MS scans was 50 ms with an AGC target of 1E5. The MS/MS precursor isolation window was set to *m/z* 4.0. Collision energy in NCE mode was used at 25/35/45. Dynamic precursor exclusion was set to 10 s.

### Data pre-processing and in silico annotation

Raw files were converted into centroid mode using MSconvert (Chambers et al., 2012). Feature finding, chromatogram deconvolution, alignment, gap filling, feature filtering and ion identity (IIN) were performed using MZmine 3.2.8, as described in Table S3 (Schmid et al., 2023). Blank-related features (cut-off value: 0.3) and features with a relative coefficient variation higher than 30% in the QC samples were removed prior to analysis. Adducts and in-source fragments were also removed to reduce the presence of overrepresented features. SIRIUS 5.0 was used for in silico compound classification using CANOPUS (CSI: FingerID) (Djoumbou Feunang et al., 2016; Dührkop et al., 2019; Duhrkop et al., 2015; Kim et al., 2021). Class assignments were annotated using the ClassyFire chemical ontology. Annotations with a probability higher than 0.5 were considered valid. Class assignments with a probability lower than 0.5 were considered invalid and changed to “Unknown”.

### Antibacterial activity

The antibacterial activity was assessed using the disk diffusion method against virulent type-reference strains of bacterial pathogens of each tree (Table S4). Briefly, 10 ml of King’s B broth (King et al., 1954) or Lysogeny (LB) broth (Composition per L, Tryptone 10 g, Yeast extract 5 g, NaCl 10 g) was inoculated with the pathogens *Pseudomonas syringae* pv. *syringae* 9644 (*Pss* 9644), *P. amydali* pv. *morsprunorum* R1 5244 (*Psm* 5244), *P. syringae* pv. *aesculi* 2250 (*Pae* 2250), *P. savastanoi* pv. *fraxini* 1006 (*Psf* 1006), *Brenneria goodwinii* FRB171 (*Bg* FRB171), *Gibbsiella quercinecans* FRB124 (*Gq* FRB124) or *Rahnella victoriana* BRK18a (*Rv* BRK18a), and incubated overnight at 27^o^C and 120 rpm. After incubation, the cell density was adjusted to 0.2, further diluted to 1:100 in KB or LB semi-solid medium (0.75% w/v agar) and poured into 90 mm Petri dishes. Simultaneously, 25 µl of each extract was added to a sterile 6 mm-paper disk and left to dry. Once the solvent evaporated, three paper disks per extract were placed in each correspondent Petri dish and incubated for 24 h at 27 °C. The diameter of observed inhibition halos was recorded, and differences between treatments were evaluated by a one-way ANOVA followed by a Tukey-Kramer Honestly Significant Differences test (*p* < 0.05). Data were analyzed using Prism 9 (GraphPad Software 9.4.1, San Diego, California, USA).

### Metabolic profiling and chemical diversity assessment

To analyze the effect of the extraction factors and their interactions on the tree extracts’ metabolic profile, a Permutational Multivariate Analysis of Variance (PERMANOVA), implemented with the *adonis2* function in the package ‘vegan’ (R 4.1.0), was performed on a Bray-Curtis dissimilarity matrix. NMDS plots of this matrix were generated to visualize the sample’s dispersion using ggplot2. To analyze group differences, a post-hoc pairwise PERMANOVA for each solvent group was performed, correcting for multiple comparisons using the *pairwise.adonis* function and the Bonferroni correction. Additional analysis of group dispersion was assessed using the *betadisper* function in the ‘vegan’ package (999 permutations) followed by a posthoc Tukey-Kramer HSD test.

To assess the extracts’ alpha diversity, feature richness (number of detected features) and Simpson diversity index were calculated with ‘vegan’ using a TIC (Total Ion Chromatogram)-normalized and mean-centred dataset per tree. Differences between treatments’ alpha diversity indices were evaluated by one-way ANOVA followed by a Tukey’s post hoc test (*p* < 0.05). Spearman correlations between the evaluated factors and the response variables, yield, antibacterial activity and alpha diversity indexes, were computed using the R package ‘corrplot’. Correlation *p*-values were adjusted with Bonferroni correction, using *p* < 0.05 as the significance threshold. Correlations (positive or negative) were considered weak if *ρ =* 0.1–0.29, moderate if *ρ* = 0.3–0.49, or strong if *ρ* > 0.5.

The variation in compound occurrences (presence/absence) was assessed using a binary matrix of each dataset where the relative abundances of the features were transformed to 1 if present or 0 if absent. From this matrix, features extracted in a specific solvent or condition were identified and visualized as Upset plots using the R package ‘ComplexUpset’. The distribution of these unique features among chemical classes was evaluated as an indicator of their diversity.

To test differences in the extracts’ chemical class abundances, a Two-way ANOVA and Tukey’s multiple comparison tests (Prism 9) were applied to a matrix in which the relative abundance of features that belonged to the same chemical class was summed, maintaining those chemical classes with a relative abundance above 0.05 as individual categories, whilst those below this threshold were grouped into a category named “other”. This analysis was complemented by unsupervised principal component analysis (PCA) and supervised orthogonal partial least-squares discriminate analyses (OPLS-DA) using SIMCA 17 (Umetrics AB, Malmö, Sweden). PCA models were generated using centred and scaled (unit-variance or Pareto scaling) data. OPLS-DA models were built using centred and Pareto-scaled data. Features with VIP scores > 1.5 were selected as discriminant variables, and their relative abundance (normalized peak area) variation was evaluated with an unpaired t-test per feature in Prism 9. False discovery rates were controlled at 1% using the two-stage linear step-up procedure of Benjamini, Krieger, and Yekutieli (Benjamini et al., 2006). R^2^X, R^2^Y and Q^2^Y scores were used to assess the models’ variance coverage and predictability. CV-ANOVA and permutation test (*n* = 999) were performed to demonstrate the significance, stability and non-randomness of the OPLS-DA models.

Finally, the data obtained using the protocol that generated the best overall results was used to compare the metabolic profiles of the four tree species. For this analysis, a matrix containing the relative abundance of each identified chemical class and subclass per tree was employed. PCA and OPLS-DA analyses were carried out, followed by a Spearman correlation analysis aimed at identifying which chemical classes could be positively correlated with the observed antibacterial activity and with the tree species discrimination.

## Results

### Solvent, temperature and the number of cycles affect the chemical composition of wood extracts

**Fig. 1:**
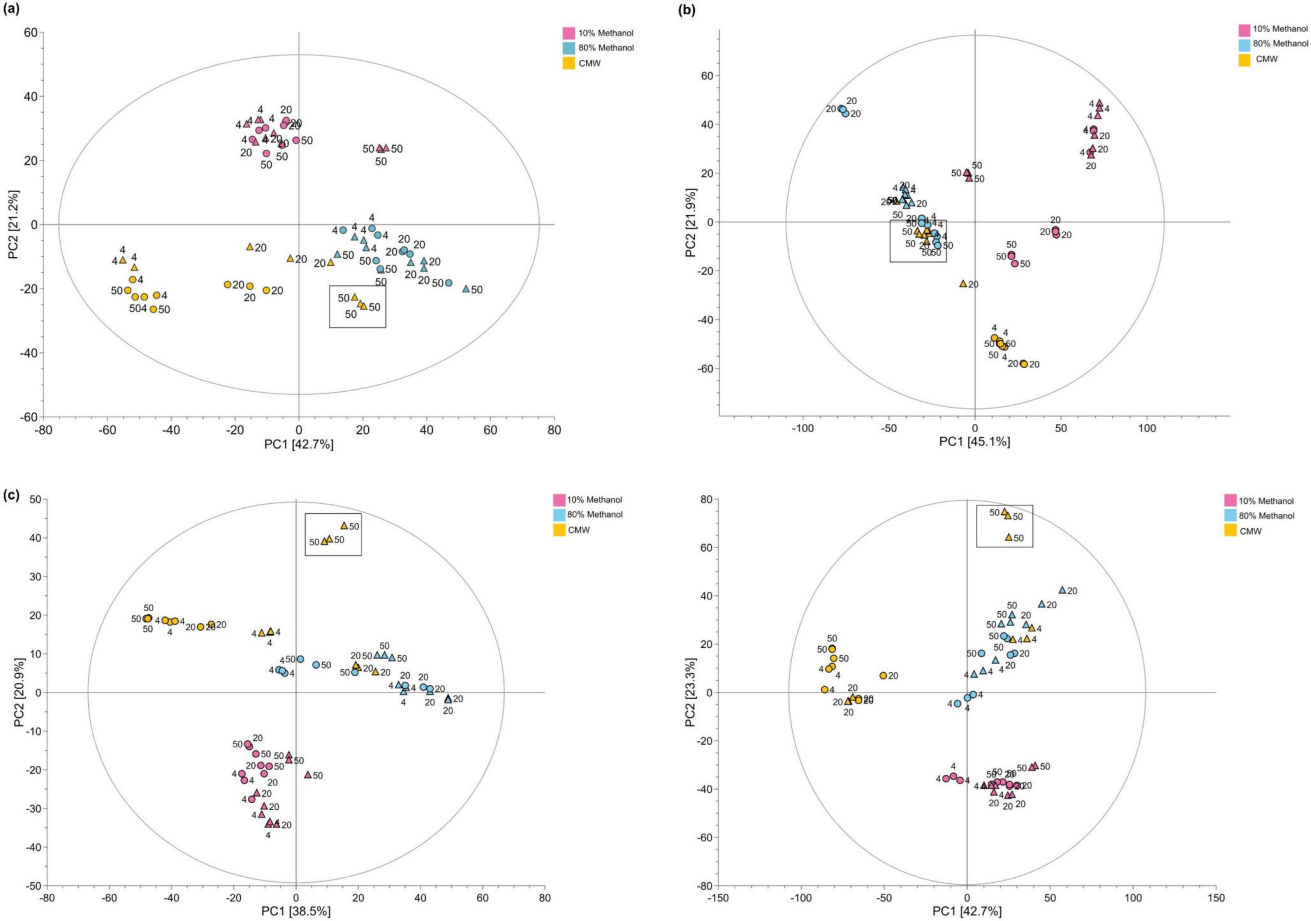
Effect of different extraction conditions on the metabolic profiles of extracts obtained from tree woody tissues. The figure presents the Principal component analysis (PCA) of the mass spectral data collected from (**a**) cherry, (**b**) ash, (**c**) horse chestnut and (**d**) oak woody tissue. The samples in the figure are coloured according to the extraction solvent (10% methanol, pink; 80% methanol, blue; CMW, orange). In the PCA, samples extracted once or three times are presented as circles or triangles, respectively. Labels on the PCA indicate the extraction temperature. P18 is highlighted with grey boxes in the score plots

PERMANOVA analysis suggested that the evaluated factors and their interaction influence the metabolic profiles of tree-wood extracts. The solvent had the most significant influence, explaining more than 50% of the datasets’ variance (Table S5), with samples primarily grouped based on the extraction solvent in the PCA (Fig. 1) and NMDS ordination plots (Fig. S1). Pairwise comparisons among solvent groups also showed significant compositional differences among the 10% (P1-P6) and 80% methanol (P7-P12) and CMW (P13-P18) groups for all the trees (Table S6). The solvent and temperature interaction also significantly affected the extracts (Table S5). Remarkably, the effect of temperature or number of cycles differed depending on the studied tree and the solvent used, as indicated by their clustering in the PCA score space, with CMW generating the highest group dispersion, particularly in the horse chestnut dataset (Table S7).

In general, 10% methanol extracts (P1-P6) tend to cluster together regardless of the studied tree (Fig. 1), with no clustering pattern observed according to temperature or number of cycles in oak and cherry. Conversely, CMW (P13-P18) and 80% methanol (P7-P12) extracts tend to cluster according to the number of extraction cycles, with P18 (CMW, 50 °C, 3 cycles) localizing further away in the PCA score space of other CMW extracts in the horse chestnut, cherry and oak datasets, suggesting that this protocol most likely leads to extracts with the most different composition in this solvent group and trees.

### Medium polarity solvents and high temperatures increase the alpha diversity of wood extracts, while the number of cycles improves the extraction yield

**Fig. 2:**
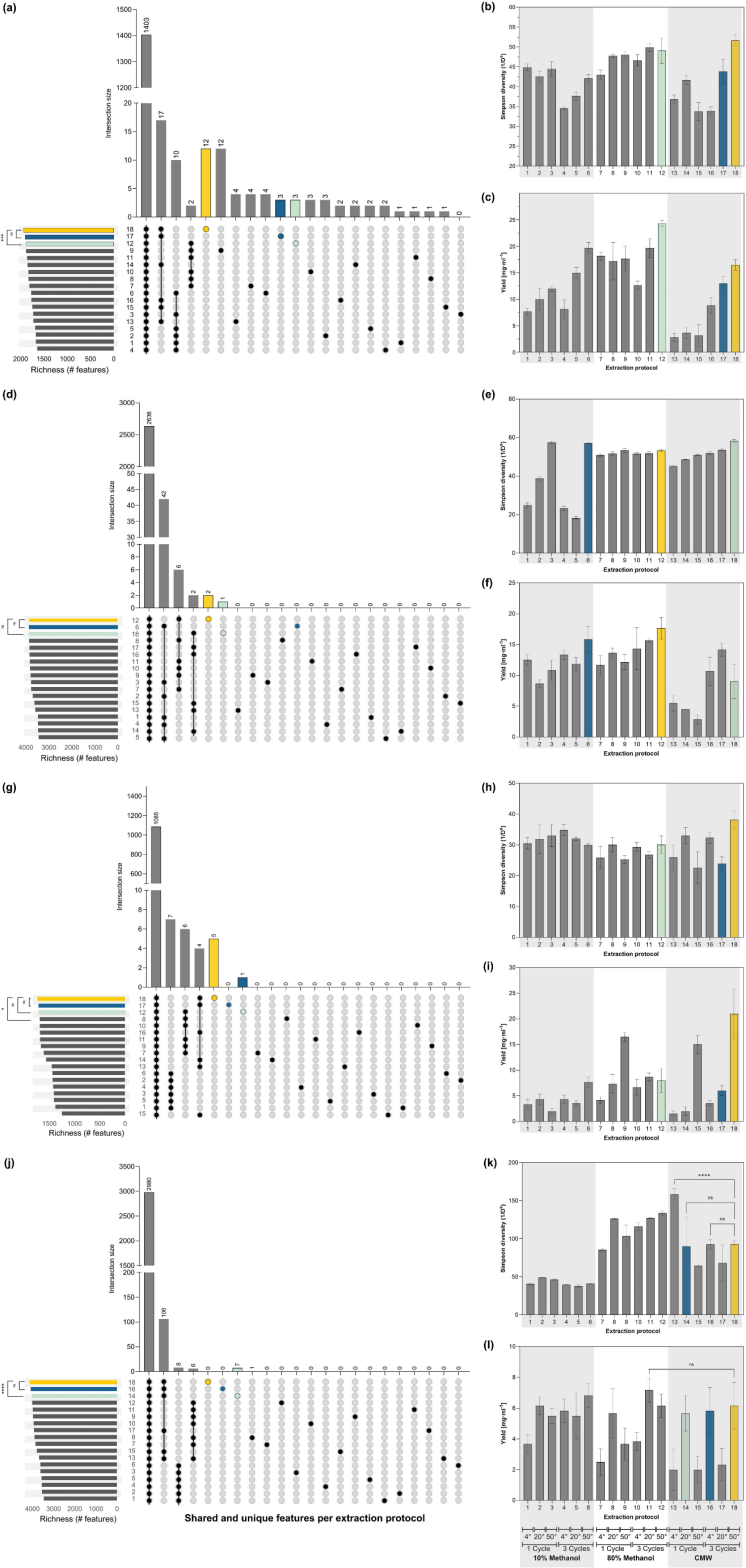
Comparative assessment of alpha diversity indexes and extraction yield across evaluated extraction protocols. The figure presents the feature richness, unique compounds, Simpson index, and extraction yield of the evaluated protocols on (**a**-**c**) cherry, (**d**-**f**) ash, (**g**-**i**) horse chestnut or (**j**-**l**) oak. The Upset plot (panels **a**, **d**, **g** and **j**) represents the unique features detected only in the specified extraction protocol (single black dots) and the shared (intersect, black line) features detected in the highlighted protocols (black dots). The bottom left bar chart shows the protocol richness (number of features detected). Error bars from the Simpson diversity index and yield plots represent the standard deviation of three replicates per protocol. Protocols with the highest richness are highlighted in orange, blue or green. Significant differences among protocols are presented, indicating the *p*-value as follows: (ns) *p* > 0.05, (*) *p* ≤ 0.05, (**) *p* ≤ 0.01, (***) *p* ≤ 0.001, (****) *p* < 0.0001

**Fig. 3:**
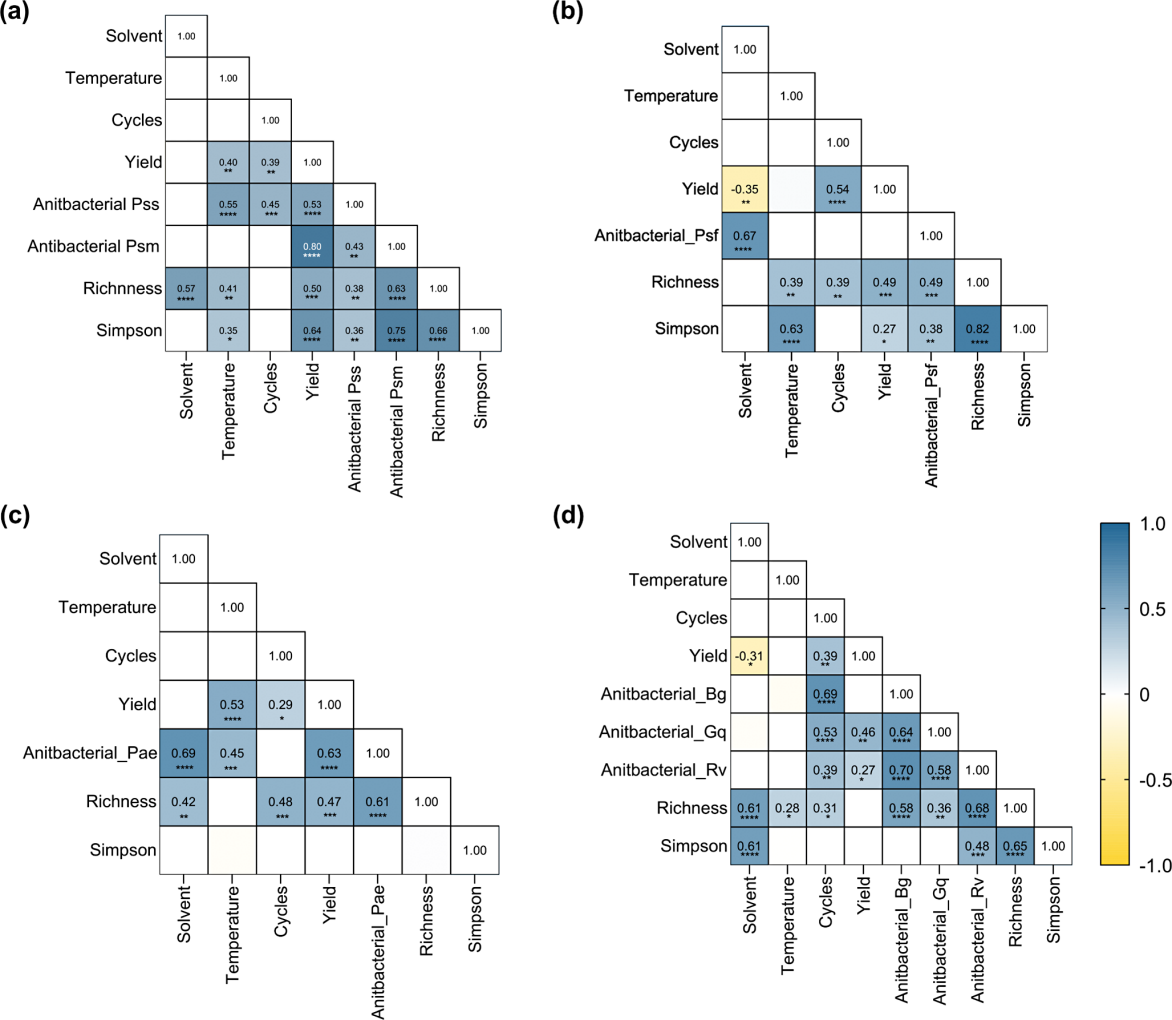
Correlation between extraction factors and the chemical diversity and antibacterial activity of wood extracts. The heatmaps represent the Spearman correlation matrix of the evaluated extraction factors and the chemical diversity indices for (**a**) cherry, (**b**) ash, (**c**) horse chestnut and (**d**) oak. The magnitude of the correlation coefficient is proportional to colour intensity according to the scale presented at the bottom right of the figure. Factors with no significant correlation were left empty. Note that correlation values were included within each cell for improved presentation. The *p*-value of the correlation is presented as follows: (*) *p* ≤ 0.05, (**) *p* ≤ 0.01, (***) *p* ≤ 0.001, (****) *p* < 0.0001

Richness and Simpson indices were calculated to evaluate if the differences in the metabolic profiles were associated with the samples’ chemical diversity. The highest feature-richness was achieved when 80% methanol and CMW were used at higher temperatures (Fig. 2), with extracts obtained with P18 having the highest richness in three out of the four tree species, along with P12 (80% methanol, 50 °C, 3 cycles). A strong positive correlation between richness-solvent was observed in cherry and oak (Fig. 3a, d). Whilst the correlation between richness-temperature was also positive, it was moderate (cherry) or weak (oak) on those trees, indicating a lower influence of the temperature on these samples richness. Similarly, in horse chestnut samples, a positive correlation between richness-solvent and richness-cycles was observed (Fig. 3c). Only in the ash dataset was the richness-solvent correlation insignificant (Fig. 3b).

The effect of the extraction conditions on the Simpson index varied among the tree species (Fig. 2b, e, h, k). In horse chestnut extracts, no significant differences were found among the protocols. In cherry, ash and oak, 80% methanol extracts (P7-P12) exhibited the highest Simpson index values, followed by CMW (P13-P18). In these trees, the Simpson index and temperature had a positive correlation (not significant in oak), indicating that increasing the extraction temperature could improve sample richness and evenness.

Although they had higher richness, the extraction yield of CMW extracts (P13-P18) was considerably lower, especially for cherry, ash, and oak (Fig. 2c, f, l). In contrast, 80% methanol (P6-P12) generates the highest yield values, with P12 allowing the recovery of a more significant amount of extract from cherry, ash and oak samples. CMW extracts yield values improved when three extraction cycles were used (P16-P18), especially in cherry and horse chestnut trees, resulting in comparable or higher yields than those obtained with 80% methanol (Fig. 2c, i). Based on these results, extraction protocols P12 and P18 were pre-selected as candidates for studying tree metabolomes and their differences in feature occurrence, chemical class abundance and diversity, and antibacterial activity were evaluated, as detailed below.

### Extraction protocols affect the presence of features from different chemical classes

During the analysis of the trees’ metabolic profiles, it was noted that 60% of the total features in every dataset were detected with all the evaluated protocols, 38% were extracted using different solvents and conditions, and less than 2% were exclusively extracted with either a specific solvent or protocol (Fig. 2a, d, g, j). The shared features among all the protocols accounted for 70% of the TIC of each sample, suggesting that the core metabolome of the trees can always be extracted regardless of the extraction conditions. Nonetheless, unique features obtained under specific conditions were observed. The highest number of unique features were obtained in CMW extracts of oak and cherry (108 and 42 features, respectively) and 10% methanol extracts of ash and horse chestnut (42 and 7, respectively). Features extracted only with specific protocols were scarce, representing less than 0.6% of the total of each dataset.

**Fig. 4:**
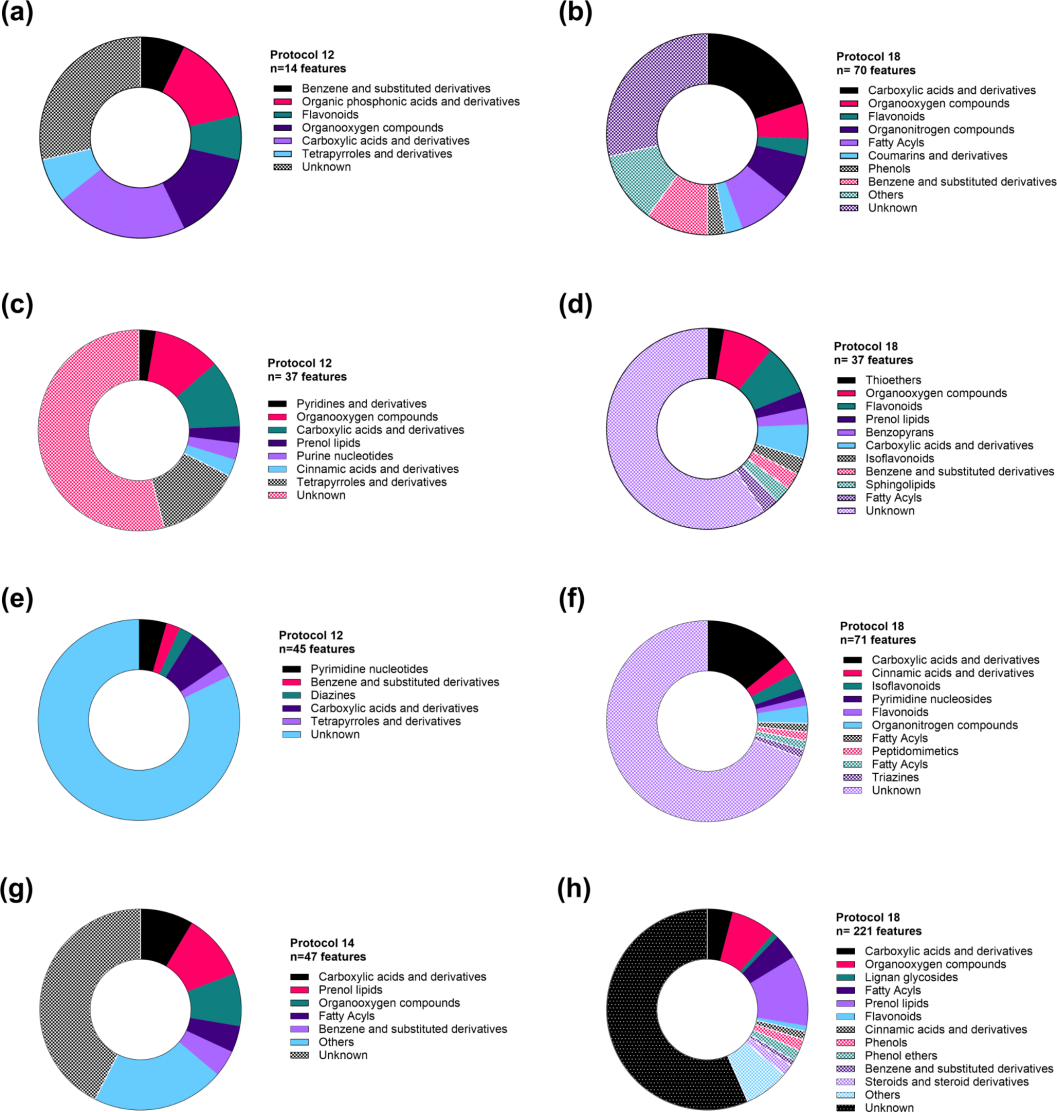
Chemical class distribution of unique features identified in the selected protocols for each tree species. The pie charts indicate the number of unique features annotated in each chemical class in the (**a**-**b**) cherry, (**c**-**d**) ash, (**e**-**f**) horse chestnut and (**g**-**h**) oak datasets. Chemical classes were annotated based on the ClassyFire chemical ontology. The number of unique metabolites identified on each protocol is presented at the top of the pie charts (n). Features whose classification score in SIRIUS was lower than 50% (probability < 0.5) and features with no classification were included in the “Unknown” group. Protocol 12–80% methanol, 50 °C, 3 cycles-; Protocol 14 -CMW, 20 °C, 1 cycles-; Protocol 18 -CMW, 50 °C, 3 cycles-

A comparison of the unique features of cherry and ash obtained using protocols P12 and P18 showed that the latter had the highest number of unique compounds distributed in sixteen chemical classes (Fig. 4a-b). In horse chestnut, P12 and P18 shared most of the features (1647 out of 1763), but P18 had 71 features not extracted in P12, and the latter had 45 unique features not observed in samples from P18 (Fig. 4e-f). In oak, P14 (instead of 12) and P18 had the best outcomes regarding alpha diversity and allowed the detection of 93% and 97% of the total number of features in the dataset. The unique features extracted with P14 (*n* = 51) belonged to 15 chemical classes (Fig. 4g), whilst those from P18 (*n* = 221) belonged to 28 chemical classes (Fig. 4h), suggesting that P18 allows the recovery of a higher number of unique features from a most diverse arrange of chemical classes.

### The relative abundance of the major wood chemical classes varies across extraction conditions

**Fig. 5:**
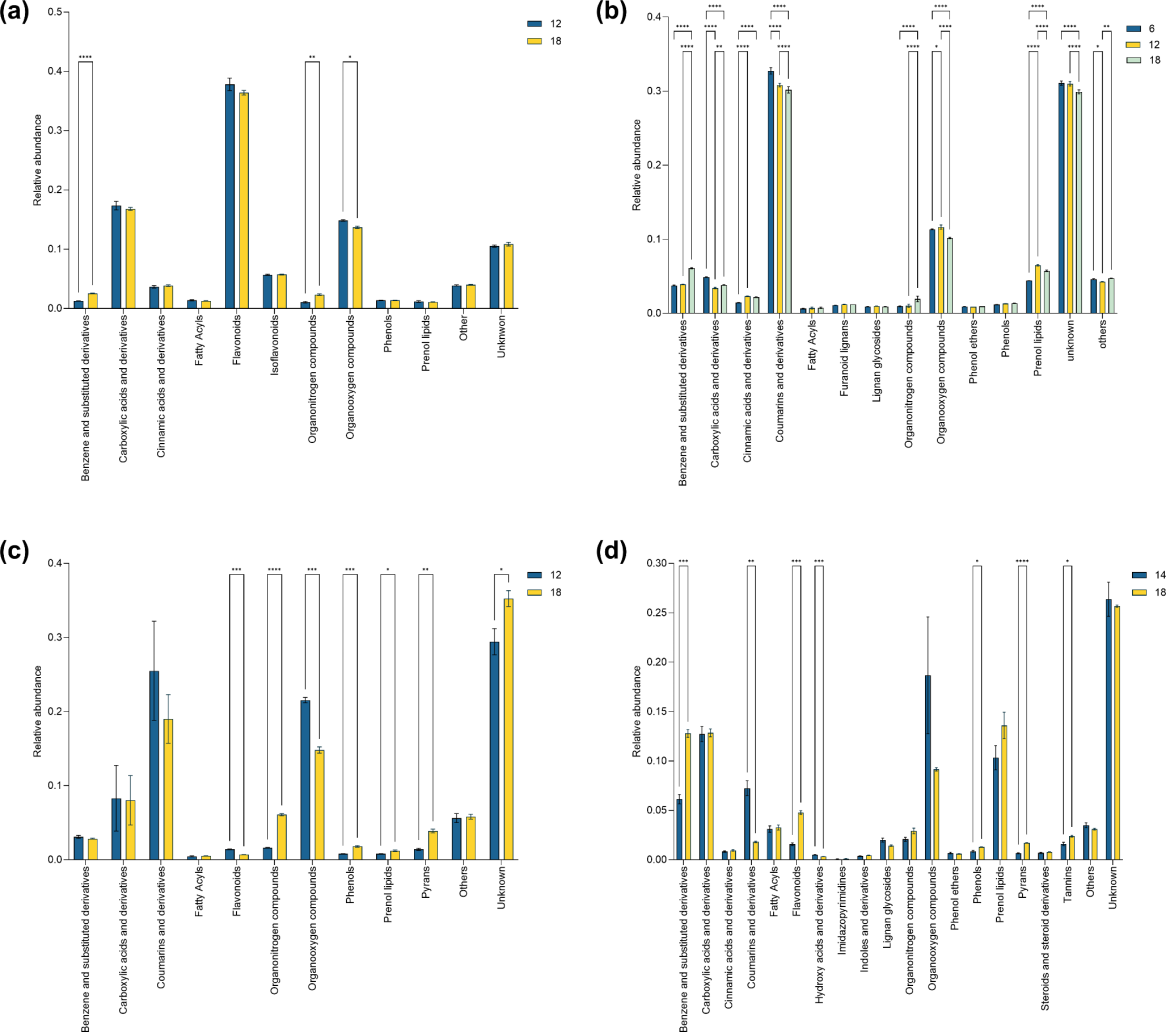
Relative abundance variation of the major chemical classes identified on each tree using selected extraction protocols. The figures illustrate the relative abundance of identified chemical taxa across individually evaluated extraction protocols for (**a**) cherry, (**b**) ash, (**c**) horse chestnut and (**d**) oak. Chemical classes whose relative abundance was lower than 0.5% were merged and presented as “Other”. Features whose classification score in SIRIUS was lower than 50% (probability < 0.5) and features with no classification were included in the “Unknown” group. Significant differences are shown as (*) *p* ≤ 0.05, (**) *p* ≤ 0.01, (***) *p* ≤ 0.001, (****) *p* < 0.0001. Protocol 12–80% methanol, 50 °C, 3 cycles-; Protocol 6–10% methanol, 50 °C, 3 cycles-; Protocol 14 -CMW, 20 °C, 1 cycles-; Protocol 18 -CMW, 50 °C, 3 cycles-

During the analysis of the trees’ metabolic profile, it was observed that the number and abundance of chemical classes varied according to the tree species and protocols, with samples extracted three times, with CMW (P16-P18) having a higher number of identified classes (Fig. 5, Fig S2). Although the main chemical classes per tree remained consistent, their relative abundance was variable (Fig. S3-S6). For example, carboxylic acids tend to be more abundant in extracts obtained with 10% methanol (P1-P6), while prenol lipids and coumarins were usually more abundant in CMW extracts (P13-P18).

In the cherry extracts, the main (more abundant) chemical classes were flavonoids, isoflavonoids, carboxylic acids, and organooxygen compounds (Fig. S3). P18 had a significantly higher abundance of benzene and substituted derivatives and organonitrogen compounds than P12. This protocol also allowed a higher recovery of cinnamic acids and isoflavonoids, although the difference was not significant. Conversely, organooxygen compounds and flavonoids were more abundant in P12 samples.

Ash extracts’ major chemical classes were benzene and substituted derivatives, carboxylic acids, coumarins, organooxygen compounds, and prenol lipids (Fig. S4). Since P6 (10% methanol, 50 °C, 3 cycles) has the second highest yield, and minor differences were observed regarding richness indices with P12 and P18, this protocol was also included in the analysis. P18 had a significantly higher relative abundance of benzene and substituted derivatives, cinnamic acids, and organonitrogen compounds. Protocol 6 showed a higher abundance of carboxylic acids and coumarins, whereas P12 had the highest abundance of organooxygen compounds and prenol lipids. Similarly, Horse chestnut extracts’ major chemical classes were benzene and substituted derivatives, carboxylic acids, coumarins, and organooxygen compounds (Fig. S5). P18 yielded a significantly higher abundance of organonitrogen compounds, phenols, prenol lipids, and pyrans. Conversely, P12 presented a higher abundance of flavonoids, organooxygen compounds, and coumarins (not significantly different from P18).

In the oak dataset, the major chemical classes were carboxylic acids, organooxygen compounds, prenol lipids, fatty acyls and flavonoids. In this dataset, there was a significant variation among the pre-selected protocols (P14 and P18) regarding the abundance of each chemical class (Fig. S6). P18’s relative abundance of benzene, flavonoids, phenols, pyrans, and tannins was higher than that observed in P14 (Fig. 5d). On the contrary, coumarins and hydroxy acids were significantly more abundant in extracts obtained with P4. Other classes, such as cinnamic acids, fatty acyls and prenol lipids, were more abundant in P18, although the difference was not significant against P14.

These results were further supported by clear discrimination between the selected protocols observed in the PCA and OPLS-DA (Fig. S7, Table S8). The features contributing to the group’s discrimination were selected based on their VIP scores from the OPLS-DA models (Fig. S8-11, Table S9). In cherry, horse chestnut, and oak datasets, P18, and P12 for ash had the highest number of upregulated (more abundant) features (Table S10). These features also belong to more chemical classes, ranging from 14 in cherry to 37 in oak. Carboxylic acids and organooxygen compounds were most frequently found within the discriminant features.

### Chemical families involved in the discrimination between tree species are correlated with the antibacterial activity against host and non-host bacterial pathogens

**Fig. 6:**
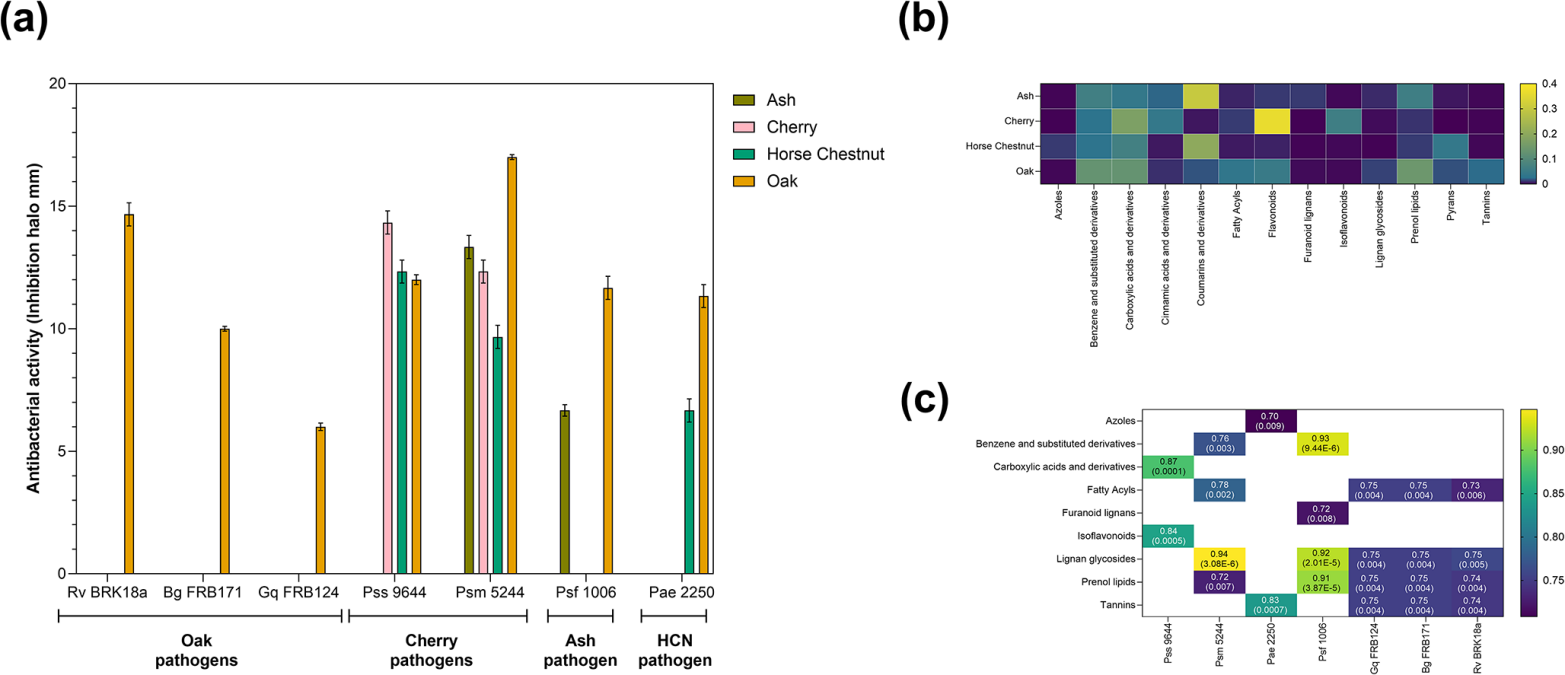
Antibacterial activity, chemical profile variation and their correlation among the four evaluated tree species. Panel (**a**) presents the average (barplot) antibacterial activity of the tree extracts obtained with P18, against seven tree-pathogenic bacteria. Error bars represent the standard deviation of three biological replicates evaluated per bacteria and tree extract. Panel (**b**) presents the relative abundance (log_10_) of the major chemical classes observed on each studied tree. Correlation analyses presented on panel (**c**) were calculated using the Spearman rank correlation method. Only discriminant chemical classes or subclasses with a strong correlation degree (Spearman correlation coefficient ρ > 0.7) and significant p-values (*p* < 0.05) were included. In panel (**c**), the Spearman correlation coefficient value is represented according to the scale colour at the bottom. For clarity, this value is also included within each cell, accompanied by the correlation p-value (presented in brackets). All the figures were created with the data acquired from the extracts obtained with P18 (CMW, 50 °C, 3 cycles). HCN, Horse chestnut

To evaluate the effect of the extraction conditions on the recovery of biologically active metabolites related to plant defence, the antibacterial activity of the 18 extracts from each tree against their cognate pathogens (oak, *Bg*, *Gq* and *Rv*; cherry, *Pss* and *Psm*; ash, *Psf*; horse chestnut (HCN), *Pae*, Fig. 6a) was determined. It was observed that the antibacterial activity is highly variable, being influenced differently for each tree by the extraction conditions. Horse chestnut and ash extracts, for example, were active against *Pae* 2250 and *Psf 1006* only when 80% methanol or CMW were used as extraction solvents (Fig. S12 b, d). Similarly, oak extracts activity against the three AOD-related pathogens (*Bg* FRB171, *Gq* FRB124 and *Rv* BRK18a) was observed on all the 80% methanolic extracts (P7-P12), and on CMW extracts obtained after three extraction cycles (P16-18). On cherry, the extraction parameters influenced the activity against *Pss* 9644 in such a way that only those samples extracted three times at 50 °C (P6, P12 and P18) were active (Fig. S12a), whilst they had no particular effect on the activity against *Psm* 5244. Overall, CMW and 80% methanol stand out as suitable solvents for the recovery of biologically active metabolites (Fig. S12), while 10% methanol is not recommended as it presented the lowest activity among the tested conditions. Still, as observed in cherry, temperature can also influence the active metabolites recovered and should be selected accordingly.

Given that P18 repeatedly showed one of the highest antibacterial activity, richness, and chemical diversity (including number of chemical classes detected and frequency of unique compounds) in most of the trees (excluding ash), the data from these extracts were used to do a comparative analysis of the trees’ metabolic profile and a correlation analysis with the antibacterial activity against host and non-host pathogens. As expected, each tree presented a particular metabolic profile characterized by the abundance of specific chemical classes (PERMANOVA F (3,8) = 549.7, *p* = 0.001, Fig. 6b) and subclasses (PERMANOVA F(3,8) = 433.8, *p* = 0.001). Out of the four trees, oak had the highest richness and Simpson index with 4062 (± 7.5) features detected with protocol 18, followed by ash (3724 ± 16.8), cherry (1875 ± 17) and horse chestnut (1714 ± 4.9) (Fig. 2; Table S11). Thirteen chemical classes and thirty-three subclasses were related to the discrimination of the studied tree species (Table S12). Notably, the number of chemical classes and subclasses identified in the oak dataset (84 and 123, respectively) were significantly lower than the ones obtained from ash (94 and 135, respectively); however, the Simpson index was significantly higher (oak: 93.11 ± 3.73, ash: 58.24 ± 0.64) suggesting that the oak wood metabolic profile is dominated by a lower number of highly abundant metabolites (Figure SI2, Table S11).

Prenol lipids were the dominant chemical class in the oak wood extracts, which has a significantly higher abundance than the other trees alongside fatty acyls, lignan glycosides and tannins (Fig. 6b). Notably, all these chemical classes were positively correlated with the antibacterial activity against the AOD pathogens, and some of them had antibacterial activity against *Psf* 1006 (prenol lipids and lignan glycosides), *Pae* 2250 (tannins), and *Psm* 5244 (prenol lipids, fatty acyls, and lignan glycosides) (Fig. 6c). In fact, oak extracts were the only ones active against the AOD pathogens (Fig. 6a) and the four *Pseudomonas*, which are not pathogenic to oak, whilst the activity of cherry, horse chestnut and ash was limited to their cognate pathogens and *Psm 5244*, suggesting that the active metabolites probably belong to one of these chemical classes, and indicating that oak has the broadest antibacterial activity across the evaluated trees.

## Discussion

During the past decade, MS-metabolic profiling of forest trees has helped to understand trees’ responses to biotic and abiotic stressing factors (Rodrigues et al., 2021). The extraction techniques used by those studies are highly heterogeneous as different approaches have been used to obtain extracts with adequate coverage of the tree metabolome (Rodrigues et al., 2019). As far as we know, no systematic analysis of the extraction protocol’s effect on the chemical diversity of wood extracts using untargeted metabolomics has been performed. Therefore, we evaluate eighteen protocols encompassing some of the most used extraction conditions in tree metabolomics to determine if varying the solvent, temperature, or number of extraction cycles could increase the chemical diversity of the extract so that a protocol with high coverage of the wood metabolome could be chosen for four model trees.

Notably, more than 60% of the features detected for each tree were obtained in all the tested protocols, accounting for 70% of the samples’ TIC, indicating that a core metabolome can be recovered regardless of the extraction conditions, similar to the results obtained by Doppler et al. (2016). Even so, the solvent significantly affected the extracts’ metabolic profile, with CMW increasing the extraction of a broader range of metabolites. An increase in the detected chemical classes has previously been reported while using combinations of polar solvents like methanol or ethanol, apolar solvents such as chloroform or acetonitrile, and water (Creydt et al., 2018; Doppler et al., 2016; Martin et al., 2014). Three-solvent mixtures also seem to improve the precision of metabolite abundance detection (measured as the Relative Standard Deviation, RSD) (Doppler et al., 2016). This effect was observed in the samples from protocol 18 as they cluster closer in the score space compared to other protocols, have fewer features with an RSD higher than 30% and presented an overall RSD lower than 10%, suggesting higher precision (Table S13). This characteristic is important in metabolomics datasets as higher precision lowers the possibility of finding false positive or negative metabolic markers (differentially expressed metabolites) while comparing sample groups (e.g. while comparing symptomatic vs. asymptomatic trees), thus improving the biological interpretation of the data (Broadhurst et al., 2018).

The effect of temperature and the number of extraction cycles was highly variable, with the number of cycles having less influence than the temperature. In general, when higher temperatures and an increasing number of extraction cycles were used, the richness and yield of the extractions were improved, particularly in CMW protocols. The effect of the number of cycles was possibly related to the solubilization of metabolites that were not extracted during the first cycle due to the solvent saturation (Bayona et al., 2018; Mosca et al., 2018), while increasing the extraction temperature may help favour the diffusion of metabolites by decreasing the solvent viscosity, increasing the solubility of the compounds, and making the matrix-analyte interaction weaker, allowing higher diffusion rates (Aguiar et al., 2022; Bayona et al., 2018; Morsli et al., 2021; Mosca et al., 2018).

High temperatures can also cause degradation of thermolabile metabolites (Pinto et al., 2022; Seidel, 2012). Thermal degradation varies depending on the class of metabolites. Most amino acids, organic acids, sugars, condensed tannins and free fatty acids, alongside some phenolic compounds and flavonoids, considered to be stable at temperatures ranging from 85 to 100 °C (Paepe et al., 2014; Fang et al., 2015; Gaugler & Grigsby, 2009). However, flavonoid glycosides, phenols, alkaloids, lipids and tannins can be degraded after being exposed to mid-high temperatures (40–60 °C) (Elnaker et al., 2021; Sung et al., 2019; Xu et al., 2013; Yu & Bulone, 2021; Yuan et al., 2022). We did not specifically evaluate thermal degradation; however, we observed that only a minority of the flavonoid glycosides and iridoid glycoside features (selected based on reports of their thermolability) in cherry and ash (trees with high abundance of these chemical classes) had lower abundance in protocols that used 50 °C (Fig. S13, S15). In fact, on the cherry dataset, P18 extracts had a higher abundance of flavonoid glycosides (Fig. S15), while the abundance of flavonoid aglycones, such as naringenin or eriodyctiol, were not modified in either the ash or cherry datasets (Fig. S14, S16), suggesting that the 50 °C extraction parameter had no significant degradation features.

As the trees’ chemical composition differed, various metabolites and chemical classes were involved in the variation of extraction protocols. Carboxylic acids, prenol lipids, flavonoids, and organooxygen compounds varied in frequency and abundance for all the trees. These metabolites dominated the classifiable set of wood metabolites alongside coumarins and cinnamic acids. Different studies have reported coumarins and prenol lipids as significant components of ash leaves and wood metabolome, highlighting their diversity within this species (Iossifova et al., 1997; Nemesio-Gorriz et al., 2020; Sidda et al., 2020). Secoiridoid glycosides especially have been of utmost interest as biological markers of susceptibility of ash trees towards *Hymenoscyphus pseudoalbidus*, the causal agent of ash dieback (Nemesio-Gorriz et al., 2020; Sidda et al., 2020; Sollars et al., 2017) and tolerance towards the emerald ash borer (Stanle et al., 2022; Villari et al., 2016).

A similar outcome was seen for oak and cherry, where phenolic compounds were the most abundant metabolites within each dataset in concordance with previous studies. In both trees, tannins, organonitrogen compounds (especially polyamines), flavonoids and hydroxycinnamic acids are the most reported and studied chemical classes due to their antioxidant, antibacterial, antifungal and anti-hyperglycemic properties (Agarwal et al., 2021; Burlacu et al., 2020; Morales, 2021; Nunes et al., 2021; Ortega-Vidal et al., 2021; Sánchez-Hernández et al., 2022; Skrypnik et al., 2019; Willig et al., 2022). The role of these metabolites on tree-pathogen interactions has also been described for fungal species, like *Blumeriella jaapi* (Rehm) *var*. Arx. (Oszmiański & Wojdyło, 2014), *Alternaria alternata* (Pan et al., 2023), or *Erysiphe alphitoides* (Griffon and Maubl.; U. Braun and S. Takam.) (Kebert et al., 2022), but their role in the tree’s defence against tree bacterial pathogens is not known. Still, they have been reported as inducers of plant resistance or antibacterial metabolites against plant bacterial pathogens such as *P. syringae* pv. *tomato* and pv. *phaesicola* (Xie et al., 2021), and human pathogens like *Pseudomonas aeruginosa*,* Escherichia coli*,* Staphylococcus aureus*, and *Candida albicans* (Abedini et al., 2020; Anlas et al., 2019; An et al., 2021; Šukele et al., 2022; Tanase et al., 2022; Yang et al., 2016). Therefore, ensuring that the selected extraction protocol had a good representation of phenolic compounds, like tannins or flavonoids, is essential as they might be involved in the tree response to the bacterial canker and AOD pathogens.

As one of the main characteristics of these metabolites is inhibiting the growth of pathogens, we examined extracts’ antibacterial activity against host and non-host bacterial pathogens to test whether all protocols resulted in the extraction of biologically relevant compounds. Interestingly, samples extracted thrice with 80% methanol or CMW at 20–50 °C (P9 and P12, and P15 and P18, respectively) tended to have higher antibacterial activity. Notably, P18 oak wood extracts displayed the broadest activity, the highest abundance of lignan glycosides, prenol lipids and tannins (positively correlated with the antibacterial activity against most of the pathogens), as well as the highest metabolite richness of the four evaluated trees. This result suggests that chemical diversity, and in particular the abundance of polyphenols, influences bioactivity, with individuals with higher richness and abundance of structurally diverse metabolites also exhibiting higher antibacterial activity.

In the *Quercus* genus for instance, the content of polyphenols has been linked to bark antibacterial activity, with species like *Q. robur*,* Q. alba* and *Q. havardii* having higher content of these chemical classes also displaying the highest antibacterial activity compared to other plants (or *Quercus*) species (Dettweiler et al., 2019; Elansary et al., 2019; Min et al., 2008). The enhanced activity may not simply correspond to an increase in the abundance of specific antimicrobial compounds, but also to a synergistic effect among the major and minor structural diverse metabolites. This phenomenon has been observed with combinations of several phytochemicals isolated from trees, like catechin and taxifolin, or protocatechuic acid, vanillic acid and catechin, resulting in higher antimicrobial activity against human (Bernal-Mercado et al., 2018) and plant pathogens (Hammerbacher et al., 2019). Synergy among chemical classes could also be playing a role in oak activity, which will be a key test in future analysis.

While this research did not determine the specific metabolites responsible for the observed antibacterial activity, it highlighted the correlation between the abundance of certain chemical families, such as lignan glycosides, prenol lipids and tannins. The ecological role of tannins has been extensively investigated due to their involvement in plant defence against herbivory (Barbehenn et al., 2005; Barbehenn & Peter Constabel, 2011). Given their antibacterial activity (Javed et al., 2020; Puljula et al., 2020) and abundance in plants challenged with different pathogens, it has been suggested that tannins may also have a role during the plant response to pathogens. Prenol lipids, on the other hand, have been associated with leaves’ tolerance to drought, salt stress, herbivory, and part of the plant response towards *Phytophthora cinnamomi* (Baczewska-Dąbrowska et al., 2022; Degenhardt et al., 2009; Macabuhay et al., 2022; Neves et al., 2023). Both chemical families are highly interesting in plant-pathogen interactions, but they have a wide variety of polarities and concentrations, making finding a suitable extraction method an analytical challenge (Aldana et al., 2020; Fraga-Corral et al., 2020; Guo et al., 2020). Here, we found that the variation of the Bligh and Dyer solvent, CMW, tends to recover a higher abundance of both chemical families in most of the studied trees, indicating that this solvent mixture is appropriate for the recovery of tannins and prenol lipids.

## Conclusion

Overall, no standard protocol could be selected for all the trees as several protocols allowed the identification of chemically diverse and active extracts. Although a “core metabolome” could be defined with most of the tested extraction protocols, 80% methanol or CMW at 50 °C for three cycles (P12 and P18) yielded the highest richness, yield and antibacterial activity in the four biological models studied; hence, these are suggested as an appropriate starting point for untargeted study of trees’ woody tissue. The extraction conditions could be further refined from these broad-spectrum protocols for targeted analysis, if a particular group of metabolites are of interest (i.e. polyphenols involved in defence mechanisms or antimicrobial compounds). This tailoring can be achieved by adjusting the extraction system to favour the recovery of the chemical class of interest. Based on the obtained data, the main factor that should be considered for these targeted analyses is the solvent, followed by the temperature. In the case of the studied trees, CMW seems to be best suited for cherry, horse chestnut and oak, while 80% methanol gave the best results for ash. Further research on the applicability of these methods for studying the tree response towards bacterial pathogens on the four evaluated tree models will be performed alongside a secondary analysis of the antibacterial activity of the crude extracts.

## Electronic Supplementary Material

Below is the link to the electronic supplementary material.

Supplementary Material 1

Supplementary Material 2

## Data Availability

Code and data for the statistical analysis of this study are available at the Zenodo repository: 10.5281/zenodo.8351670 (https://doi.org/10.5281/zenodo.8351670). Raw and mzML data from mass spectrometry are publicly available in the MassIVE repository and can be downloaded with the MassIVE ID MSV000092816.
