# Supplementary material for: Metabolic profiling and antibacterial activity of tree wood extracts obtained under variable extraction conditions
Source: Metabolomics. 2024 Dec 27;21(1):13. doi: 10.1007/s11306-024-02215-x (PMC11680671; doi:10.1007/s11306-024-02215-x)
Supplement: Supplementary file 1 — Supplementary Material 1 [file 11306_2024_2215_MOESM1_ESM.docx]

**Metabolic profiling and antibacterial activity of tree wood extracts obtained under variable extraction conditions**

**Supplementary Information**

Diana Vinchira-Villarraga^1^, Sabrine Dhaouadi^1^, Vanja Milenkovic^1^, Jiaqi Wei^1^, Emily R. Grace^1^, Katherine G. Hinton^1^, Amy J. Webster^1^, Andrea Vadillo-Dieguez^1^, Sophie E. Powell^1^, Naina Korotania^1^, Leonardo Castellanos^2^, Freddy A. Ramos^2^, Richard J Harrison^3^, Mojgan Rabiey^1,4^ & Robert W. Jackson^1^

^1^ School of Biosciences and the Birmingham Institute of Forest Research, University of Birmingham, Birmingham B15 2TT, UK

^2^ Facultad de Ciencias, Departamento de Química, Universidad Nacional de Colombia - Sede Bogotá, Bogotá, Carrera 30# 45-03, Bogotá, D.C. 111321, Colombia.

^3^ Plant Sciences group, Wageningen University & Research, Wageningen 6700AA, The Netherlands.

^4^School of Life Sciences, Gibbet Hill Campus, University of Warwick, Coventry CV4 7AL, UK

**Table S1. Description and location of the trees used in the present study.**

| Tree | Tree ID | Diameter at breast height (cm) | Location (GPS) | Health status (Symptoms of bacterial disease) | UoB_ID number |
| --- | --- | --- | --- | --- | --- |
| Oak (*Quercus spp.*)^2^ | 1 | 239 | 52.448551, -1.931853 | None | 00269 |
|  | 2 | 292 | 52.448821, -1.925759 | None | 008595 |
|  | 3 | 252 | 52.448747, -1.925912 | None | 008594 |
|  | 4 | 244 | 52.449712, -1.930217 | None | NA |
|  | 5 | 170 | 52.449532, -1.931132 | None | 00533 |
| Cherry (*Prunus avium*) | 1 | 72 | 52.450619, -1.932467 | Bacterial canker symptoms^1^ | 00631 |
|  | 2 | 79 | 52.450697, -1.932467 | None | 07130 |
|  | 3 | 207 | 52.449084, -1.932893 | None | 06345 |
|  | 4 | 218 | 52.448901, -1.933054 | Bacterial canker symptoms^1^ | NA |
|  | 5 | 94 | 52.448709, -1.9335480 | None | 00065 |
| Ash (*Fraxinus excelsior*) | 1 | 28 | 52.451352, -1.9342780 | None | 06945 |
|  | 2 | 29 | 52.451352, -1.9341780 | None | 06944 |
|  | 3 | 30.6 | 52.451352, -1.9342780 | None | 06943 |
|  | 4 | 32 | 52.451372, -1.9339186 | None | 06942 |
|  | 5 | 34 | 52.451357, -1.934079 | None | 06941 |
| Horse chestnut (*Aesculus hippocastanum*) | 1 | 285 | 52.4480290, -1.9249842 | None | 03926 |
|  | 2 | 278 | 52.4479312, -1.9248407 | None | 03927 |
|  | 3 | 204 | 52.4481083, -1.9245906 | None | 03930 |
|  | 4 | 165 | 52.453356, -1.929137 | None | 5676 |
|  | 5 | 262 | 52.45810, -1.929508 | None | 07370 |

^1^ In these individuals, evidence of a past infection of bacterial canker was observed. These symptoms corresponded to callus tissue with dried gum near the site of the healed canker in one (tree 4) or two (tree 1) shoots. No other parts of the trees presented symptoms, suggesting no active infection. Therefore, the trees were considered to be in remission and included in the study. Tissue was sampled >20 cm from the areas of previous infection.

^2^ The sampled oak trees corresponded to *Quercus robur* (Tree 1-4) and *Q. cerris* (Tree 5).

**Table S2. Experimental design for extracting metabolites from ash, cherry, horse chestnut and oak wood.**

| Factor | A1 | A2 | A3 | B1 | B2 | C1 | C2 | C3 |
| --- | --- | --- | --- | --- | --- | --- | --- | --- |
| P1 | + | - | - | + | - | + | - | - |
| P2 | + | - | - | + | - | - | + | - |
| P3 | + | - | - | + | - | - | - | + |
| P4 | + | - | - | - | + | + | - | - |
| P5 | + | - | - | - | + | - | + | - |
| P6 | + | - | - | - | + | - | - | + |
| P7 | - | + | - | + | - | + | - | - |
| P8 | - | + | - | + | - | - | + | - |
| P9 | - | + | - | + | - | - | - | + |
| P10 | - | + | - | - | + | + | - | - |
| P11 | - | + | - | - | + | - | + | - |
| P12 | - | + | - | - | + | - | - | + |
| P13 | - | - | + | + | - | + | - | - |
| P14 | - | - | + | + | - | - | + | - |
| P15 | - | - | + | + | - | - | - | + |
| P16 | - | - | + | - | + | + | - | - |
| P17 | - | - | + | - | + | - | + | - |
| P18 | - | - | + | - | + | - | - | + |

Factor abbreviations are presented as follows: A1: 10% Methanol; A2: 80% Methanol; A3: CMW; B1: 1 cycle; B2: 3 cycles; C1: 4°C; C2: 20°C; and C3: 50°C. within the table, a + sign indicates which condition (factor) was used in each experiment.

**Table S3. MzMine processing parameters.** Data pre-processing was done using MzMine 3.2.8

| **Dataset** | **Cherry** | **Ash** | **Horse-chestnut** | **Oak UoB and NPE** |
| --- | --- | --- | --- | --- |
| Mass detection level | MS1 | MS1 | MS1 | MS1 |
| Algorithm | Centroid | Centroid | Centroid | Centroid |
| Noise | 2E4 | 2E4 | 2E4 | 1E4 |
| Mass detection level | MS2 | MS2 | MS2 | MS2 |
| Algorithm | Centroid | Centroid | Centroid | Centroid |
| Noise | 1.5E3 | 2E2 | 2E3 | 1E3 |
| **ADAP chromatogram builder** | | | | |
| Scans | Rt: 0.5-20 min | RT: 0.5-13 min | RT: 0.5-17 min | RT: 0.5-17 min |
| Min group size # scans | 5 | 5 | 5 | 5 |
| Group intensity threshold | 8E4 | 5E4 | 4E4 | 8E4 |
| Min highest intensity | 2.5E5 | 1E5 | 9E5 | 2E5 |
| m/z tolerance | 0.003 | 0.005 | 0.005 | 0.002 |
| ppm tolerance | 5 | 10 | 5 | 5 |
| **Chromatogram deconvolution** | | | | |
| Algorithm | Local minimum resolver | Local minimum resolver | Local minimum resolver | Local minimum resolver |
| MS/Ms scan pairing | MS1 to MS2 precursor tolerance: 0.005 *m/z* or 10 ppm  Minimum relative feature height: 20%  Minimum required signals:  2 | MS1 to MS2 precursor tolerance: 0.005 *m/z* or 10 ppm  Minimum relative feature height: 20%  Minimum required signals:  1 | MS1 to MS2 precursor tolerance: 0.005 *m/z* or 10 ppm  Minimum relative feature height: 20%  Minimum required signals:  1 | MS1 to MS2 precursor tolerance: 0.005 *m/z* or 10 ppm  Minimum relative feature height: 20%  Minimum required signals:  1 |
| Chromatographic threshold | 90 | 89 % | 85% | 89% |
| Min search range RT | 0.05 | 0.04 | 0.05 | 0.04 |
| Min relative height | 1% | 1% | 1% | 0.8% |
| Min absolute height | 2.5E5 | 5E4 | 2E4 | 4E4 |
| Min ratio peak top/edge | 1.77 | 1.9 | 1.55 | 1.9 |
| Peak uration range | 0.01-2 | 0.01-3 | 0.05-3 | 0.01-3 |
| Min # data point | 5 | 5 | 5 | 5 |
| **Isotoping (13C isotope filter)** | | | | |
| m/z tolerance | 0.002 | 0.0015 | 0.002 | 0.002 |
| ppm tolerance | 5 | 3 | 5 | 5 |
| RT tolerance | 0.02 | 0.02 min | 0.02 | 0.02 |
| Max charge | 2 | 2 | 2 | 2 |
| Representative isotope | Most intense | Most intense | Most intense | Most intense |
| **Isotopic peaks finder** | | | | |
| Chemical elements | H, C, N, O, S, P | H, C, N, O, S, P | H, C, N, O, S, P | H, C, N, O, S, P |
| m/z tolerance | 0.002 | 0.002 | 0.005 | 0.002 |
| ppm tolerance | 5 | 3 | 3 | 5 |
| Max. charge of isotope m/z | 2 | 2 | 2 | 2 |
| Search in scans | Single most intense | Single most intense | Single most intense | Single most intense |
| **Alignment (Join aligner)** | | | | |
| m/z tolerance | 0.005 | 0.005 | 0.002 | 0.002 |
| ppm tolerance | 10 | 10 | 5 | 5 |
| weigth m/z | 3 | 3 | 3 | 3 |
| RT tolerance | 0.08 | 0.05 | 0.1 | 0.1 |
| Weigth RT | 2 | 1 | 1 | 1 |
| Others | Require same charge state | Require same charge state | Require same charge state | Require same charge state |
| **Filtering I** | | | | |
| Filter mode | Duplicate peak filter | Duplicate peak filter | Duplicate peak filter | Duplicate peak filter |
| m/z tolerance | 0.005 | 0.005 | 0.005 | 0.005 |
| ppm tolerance | 5 | 5 | 5 | 5 |
| RT tolerance | 0.05 | 0.05 | 0.1 | 0.05 |
| **Gap filling** | | | | |
| Intensity tolerance | 5% | 5% | 5% | 5% |
| m/z tolerance | 0.005 | 0.005 | 0.002 | 0.002 |
| ppm tolerance | 5 | 5 | 5 | 5 |
| RT tolerance | 0.07 | 0.05 | 0.1 | 0.1 |
| Min data points | 5 | 5 | 5 | 5 |
| **Filtering II (Feature filter)** | | | | |
| Duration | 0 – 3 | 0 – 3 | 0 – 3 | 0 – 3 |
| # data points | 3 – 10000 | 3 – 10000 | 3 – 10000 | 3 – 10000 |
| **Filtering III** | | | | |
| Min peaks in a row | 3 | 3 | 3 | 3 |
| Never remove peaks with M2 | Ok | Ok | Ok | Ok |
| **Metacorrelate IIN** | | | | |
| RT tolerance | 0.05 min | 0.05 min | 0.1 min | 0.1 min |
| Min height | 2E5 | 9E5 | 9E5 | 6E4 |
| Intensity correlation threshold | 5E4 | 2E4 | 2E4 | 1E4 |
| Min samples filter | Default | Default | Default | Default |
| Correlation grouping | Min data points: 5  Min data points on edge: 2  Measure: Pearson  Min feature shape correlation: 90% | Min data points: 5  Min data points on edge: 2  Measure: Pearson  Min feature shape correlation: 90% | Min data points: 5  Min data points on edge: 2  Measure: Pearson  Min feature shape correlation: 90% | Min data points: 5  Min data points on edge: 2  Measure: Pearson  Min feature shape correlation: 90% |
| Feature height correlation | Minimum samples: 3  Measure: Pearson  Min correlation: 80% | Minimum samples: 3  Measure: Pearson  Min correlation: 80% | Minimum samples: 3  Measure: Pearson  Min correlation: 80% | Minimum samples: 3  Measure: Pearson  Min correlation: 80% |
| **IIN** | | | | |
| m/z tolerance | 0.002 | 0.0015 | 0.0015 | 0.0015 |
| ppm tolerance | 5 | 3 | 3 | 3 |
| check | Average | Average | Average | Average |
| IIN library | MS mode: Positive  Maximum charge: 2  Maximum molecules/cluster:3  Adducts: [M+H]+, [M+Na]+, [M+K]+, [M+NH4]+, [M+2H]+  Modifications: [M-H2O], [M+HFA]+ | MS mode: Positive  Maximum charge: 2  Maximum molecules/cluster:3  Adducts: [M+H]+, [M+Na]+, [M+K]+, [M+NH4]+, [M+2H]+  Modifications: [M-H2O], [M+HFA]+ | MS mode: Positive  Maximum charge: 2  Maximum molecules/cluster:3  Adducts: [M+H]+, [M+Na]+, [M+K]+, [M+NH4]+, [M+2H]+  Modifications: [M-H2O], [M+HFA]+ | MS mode: Positive  Maximum charge: 2  Maximum molecules/cluster:3  Adducts: [M+H]+, [M+Na]+, [M+K]+, [M+NH4]+, [M+2H]+  Modifications: [M-H2O], [M+HFA]+ |
| Annotation refinement | Minimum size: 2 | Minimum size: 2 | Minimum size: 2 | Minimum size: 2 |

**Table S4. Bacterial pathogen used in this study with host, isolator and recent reference.**

| **Strain ID** | **Species** | **Plant host** | **Isolator** | **Reference** |
| --- | --- | --- | --- | --- |
| *Pss* 9644 | *Pseudomonas syringae* pv. *syringae* | *Prunus avium* (cherry) | Roberts, 2012 | Hulin *et al.,* 2018 |
| *Psm* 5244 | *Pseudomonas amygdali pv. morsprunorum* | *Prunus avium* (cherry) | Crosse, 1960 | Hulin *et al.,* 2018 |
| *Psf* 1006 | *Pseudomonas savastanoi* pv. *fraxini* 1006 | *Fraxinus excelsior* (ash) | J. D. Janse, 1981 | Moreno-Perez *et al.*, 2020 |
| *Pae* 2250 | *Pseudomonas syringae* pv*. aesculi* | *Aesculus hippocastanum* (horse chestnut) | Green, 2008 | Green *et al.,* 2010 |
| *Bg* FRB171 | *Brenneria goodwinii* | *Quercus robur,*  *Q. petraea* (oak) | Brady, 2009 | Denman *et al.,* 2012 |
| *Gq* FRB124 | *Gibbsiella quercinecans* | *Quercus robur,*  *Q. petraea* (oak) | Brady, 2009 | Brady *et al.,* 2010 |
| *Rv* BRK18a | *Rahnella victoriana* | *Quercus robur,*  *Q. petraea* (oak) | Brady, 2009 | Brady *et al.,* 2014 |

**Table S5. Permutational multivariate analysis of variance (PERMANOVA) testing the effect of solvent, temperature and the number of cycles on the samples on the wood metabolome.**

| **Cherry dataset** | | | | | |
| --- | --- | --- | --- | --- | --- |
| Factors | df | SS | R2 | F | Pr(>F) |
| solvent | 2 | 2.635216 | 0.530863 | 186.2081 | **<0.0001** |
| cycles | 1 | 0.202322 | 0.040758 | 28.5927 | **<0.0001** |
| temperature | 2 | 0.464962 | 0.093666 | 32.85491 | **<0.0001** |
| solvent:cycles | 2 | 0.261572 | 0.052694 | 18.48308 | **<0.0001** |
| solvent:temperature | 4 | 0.627868 | 0.126484 | 22.18303 | **<0.0001** |
| cycles:temperature | 2 | 0.229658 | 0.046264 | 16.22794 | **<0.0001** |
| solvent:cycles:temperature | 4 | 0.287688 | 0.057955 | 10.16424 | **<0.0001** |
| Residual | 36 | 0.254736 | 0.051316 | NA | NA |
| Total | 53 | 4.964023 | 1 | NA | NA |
| **Ash dataset** | | | | | |
| Factors | df | SS | R2 | F | Pr(>F) |
| solvent | 2 | 2.587332 | 0.697225 | 1556.556 | **<0.0001** |
| cycles | 1 | 0.074255 | 0.02001 | 89.34509 | **<0.0001** |
| temperature | 2 | 0.248913 | 0.067076 | 149.7477 | **<0.0001** |
| solvent:cycles | 2 | 0.132072 | 0.03559 | 79.45523 | **<0.0001** |
| solvent:temperature | 4 | 0.538139 | 0.145016 | 161.8738 | **<0.0001** |
| cycles:temperature | 2 | 0.031303 | 0.008435 | 18.83197 | **<0.0001** |
| solvent:cycles:temperature | 4 | 0.068966 | 0.018585 | 20.74529 | **<0.0001** |
| Residual | 36 | 0.02992 | 0.008063 | NA | NA |
| Total | 53 | 3.7109 | 1 | NA | NA |
| **Horse chestnut dataset** | | | | | |
| Factors | df | SS | R2 | F | Pr(>F) |
| solvent | 2 | 1.585249 | 0.513386 | 88.9101 | **<0.0001** |
| cycles | 1 | 0.104379 | 0.033803 | 11.7084 | **<0.0001** |
| temperature | 2 | 0.169679 | 0.054951 | 9.516616 | **<0.0001** |
| solvent:cycles | 2 | 0.247294 | 0.080087 | 13.8697 | **<0.0001** |
| solvent:temperature | 4 | 0.426826 | 0.138228 | 11.96947 | **<0.0001** |
| cycles:temperature | 2 | 0.081973 | 0.026547 | 4.597514 | **0.0002** |
| solvent:cycles:temperature | 4 | 0.151494 | 0.049062 | 4.248351 | **<0.0001** |
| Residual | 36 | 0.320936 | 0.103936 | NA | NA |
| Total | 53 | 3.087832 | 1 | NA | NA |
| **Oak dataset** | | | | | |
| Factors | df | SS | R2 | F | Pr(>F) |
| solvent | 2 | 2.310301 | 0.651333 | 619.8263 | **<0.0001** |
| cycles | 1 | 0.12063 | 0.034009 | 64.72702 | **<0.0001** |
| temperature | 2 | 0.208377 | 0.058747 | 55.90518 | **<0.0001** |
| solvent:cycles | 2 | 0.220709 | 0.062223 | 59.21354 | **<0.0001** |
| solvent:temperature | 4 | 0.289919 | 0.081736 | 38.89092 | **<0.0001** |
| cycles:temperature | 2 | 0.109273 | 0.030807 | 29.31674 | **<0.0001** |
| solvent:cycles:temperature | 4 | 0.220736 | 0.062231 | 29.61037 | **<0.0001** |
| Residual | 36 | 0.067092 | 0.018915 | NA | NA |
| Total | 53 | 3.547037 | 1 | NA | NA |

* Values indicated in bold are considered significant (p-value<0.05).

**Table S6. Pairwise PERMANOVA analysis of the obtained extracts for cherry, ash, horse-chestnut and oak datasets grouped by solvent.** Permutation=999, Pairwise analysis was performed using a Bray-Curtis dissimilarity matrix as input for each dataset with Bonferroni adjustment for repeated measures.

| **Cherry dataset** | | | | | | | |
| --- | --- | --- | --- | --- | --- | --- | --- |
| Group 1 | Group 2 | Df | SS | F-model | R2 | p-value | p-adjusted |
| 10% | 80% | 1 | 0.4178857 | 79.87297 | 0.701421 | 0.0001 | 0.0003 |
| 10% | MCH | 1 | 0.6403894 | 53.67824 | 0.612218 | 0.0001 | 0.0003 |
| 80% | MCH | 1 | 0.3458570 | 35.02603 | 0.507432 | 0.0001 | 0.0003 |
| **Ash dataset** | | | | | | | |
| Group 1 | Group 2 | Df | SS | F-model | R2 | p-value | p-adjusted |
| 10% | 80% | 1 | 1.8251508 | 67.62004 | 0.665420 | 0.0001 | 0.0003 |
| 10% | MCH | 1 | 1.8332464 | 59.43033 | 0.636092 | 0.0001 | 0.0003 |
| 80% | MCH | 1 | 1.8332464 | 26.96900 | 0.442339 | 0.0001 | 0.0003 |
| **Horse chestnut dataset** | | | | | | | |
| Group 1 | Group 2 | Df | SS | F-model | R2 | p-value | p-adjusted |
| 10% | 80% | 1 | 0.7483471 | 36.81989 | 0.519908 | 0.0001 | 0.0003 |
| 10% | MCH | 1 | 1.0508249 | 29.49151 | 0.464495 | 0.0001 | 0.0003 |
| 80% | MCH | 1 | 0.5787019 | 17.84396 | 0.344186 | 0.0001 | 0.0003 |
| **Oak dataset** | | | | | | | |
| Group 1 | Group 2 | Df | SS | F-model | R2 | p-value | p-adjusted |
| 10% | 80% | 1 | 0.6869196 | 98.0828 | 0.74258 | 0.0001 | 0.0003 |
| 10% | MCH | 1 | 1.8564090 | 58.7752 | 0.63352 | 0.0001 | 0.0003 |
| 80% | MCH | 1 | 0.9221227 | 26.9935 | 0.44256 | 0.0001 | 0.0003 |

**Table S7. BETADISPER and Tukey HSD analysis of the obtained extracts for the horse-chestnut dataset grouped by solvent.**

|  | Df | SS | R2 | F | p-value |
| --- | --- | --- | --- | --- | --- |
| Groups (Solvent) | 2 | 0.055779 | 0.0278894 | 10.519 | 0.001 |
| Residuals | 51 | 0.135222 | 0.0026514 |  |  |
| **Tukey HSD** | **Diff** | **Lwr** | **Upr** | **p-adj** |  |
| 10%-80% | -0.01963730 | -0.06107080 | 0.02179621 | 0.49 |  |
| 10%-CMW | 0.05620422 | 0.01477071 | 0.09763772 | 0.0053 |  |
| 80%-CMW | 0.07584152 | 0.03440801 | 0.11727502 | 0.00015 |  |

**Table S8. Generated PCA and OPLS-DA models of the cherry, ash, horse-chestnut and oak wood dataset matrix.**

| **Dataset** | **Model quality and description** | | | | | | | | | | |
| --- | --- | --- | --- | --- | --- | --- | --- | --- | --- | --- | --- |
|  | **PCA** | | | | **OPLS-DA** | | | | | **Permutation (n=999)** | |
|  | **Scaling method** | **#PC** | **R^2^X (cum)** | **Q^2^ (cum)** | **Scaling method** | **R^2^X (cum)** | **R^2^Y (cum)** | **Q^2^ (cum)** | **CV-ANOVA p-value** | **R^2^** | **Q^2^** |
| Cherry* | UV | 7 | 0.813 | 0.72 |  |  |  |  |  |  |  |
| Cherry 12 Vs 18 | Pareto | 3 | 0.957 | 0.746 | Pareto | 0.908 | 0.998 | 0.991 | 0.013 | (0.0, 0.69) | (0.0,-1.41) |
| Ash* | UV | 9 | 0.88 | 0.811 |  |  |  |  |  |  |  |
| Ash 12 Vs 18 | Pareto | 4 | 0.962 | 0.742 | Pareto | 0.805 | 1 | 0.991 | 0.014 | (0.0, 0.90) | (0.0,-2.98) |
| Horse chestnut* | UV | 7 | 0.832 | 0.74 |  |  |  |  |  |  |  |
| Horse chestnut 12 Vs 18 | Pareto | 3 | 0.931 | 0.699 | Pareto | 0.817 | 0.99 | 0.988 | 0.018 | (0.0, 0.75) | (0.0,-1.12) |
| Oak* | UV | 9 | 0.87 | 0.794 |  |  |  |  |  |  |  |
| Oak 14 Vs 18 | Pareto | 2 | 0.959 | 0.908 | Pareto | 0.958 | 0.99 | 0.995 | 0.007 | (0.0, 0.73) | (0.0,-0.55) |

*This model contains the data for all the extraction protocols (1-18) without the QC samples.

**Table S10. Important features identified for each dataset based on the OPLS-DA model.**

| **Dataset** | **Cherry** | **Ash** | **Horse chestnut** | **Oak** |
| --- | --- | --- | --- | --- |
| **Compared protocols** | 12 vs 18 | 12 vs 18 | 12 vs 18 | 14 vs 18 |
| **Selected protocol** | 18 | 12 | 18 | 18 |
| **VIP > 1.5** | 208 | 231 | 183 | 509 |
| **t-test p-value <0.05** | 101 | 162 | 144 | 498 |
| **Upregulated features*** | 52 | 115 | 100 | 459 |
| **# Chemical classes identified** | 14 | 21 | 20 | 37 |
| **Most frequent chemical class (n)^Ɨ^** | Carboxylic acids (8) Organooxygen compounds (5) | Organooxygen compounds (22) Prenol lipids (16) | Carboxylic acids (8) Phenols (8) | Prenol lipids (79) Organooxygen compounds (45) |
| **Downregulated features** | 49 | 47 | 44 | 39 |
| **# Chemical classes identified** | 10 | 13 | 12 | 12 |
| **Most frequent chemical class (n) ^Ɨ^** | Carboxylic acids (17) Organooxygen compounds (6) | Carboxylic acids (10) Organonitrogen compounds (7) | Organooxygen compounds (7) Flavonoids (6) | Prenol lipids (10) Organooxygen compounds (5) |

*Upregulated or downregulated refers to features with higher or lower relative abundance in the selected protocol.

**^Ɨ^** (n) represents the number of features within the described chemical class. Chemical classes correspond to the Class level (ClassyFire).

**Table S11. Chemical diversity of the wood extracts from the four tree species evaluated**. The data presented in the table correspond to the results obtained with protocol 18 (CMW, 50C, 3 cycles). For each parameter, the average value ± standard deviation is presented.

| **Tree** | **Sample ID** | **Richness** | **Mean Richness** | **Mean Simpson** | **Mean Simpson** | **Chemical class frequency** | **Mean class frequency** | **Chemical subclass frequency** | **Mean Subclass frequency** |
| --- | --- | --- | --- | --- | --- | --- | --- | --- | --- |
| Ash | A18_1 | 3721 | 3724.67±  16.80 | 57.92 | 58.25±  0.64 | 95 | 94.0±  0.82 | 136 | 135.0±  0.82 |
|  | A18_2 | 3710 |  | 59.15 |  | 93 |  | 135 |  |
|  | A18_3 | 3743 |  | 57.68 |  | 94 |  | 134 |  |
| Cherry | C18_1 | 1857 | 1875.00±  17.09 | 50.33 | 51.68±  1.06 | 73 | 74.0±  0.82 | 136 | 135.3±  0.94 |
|  | C18_2 | 1877 |  | 52.91 |  | 74 |  | 134 |  |
|  | C18_3 | 1891 |  | 51.81 |  | 75 |  | 136 |  |
| Horse chestnut | H18_1 | 1712 | 1714.33±  4.93 | 41.28 | 38.17±  2.24 | 75 | 74.7±  0.47 | 108 | 107.7±  1.25 |
|  | H18_2 | 1711 |  | 37.15 |  | 75 |  | 109 |  |
|  | H18_3 | 1720 |  | 36.09 |  | 74 |  | 106 |  |
| Oak | O18_1 | 4068 | 4062.67±  6.18 | 95.25 | 93.11±  3.73 | 84 | 84.0±  0 | 124 | 123.7±  0.47 |
|  | O18_2 | 4066 |  | 96.22 |  | 84 |  | 123 |  |
|  | O18_3 | 4054 |  | 87.86 |  | 84 |  | 124 |  |

**Table S12. Significant chemical classes and subclasses (VIP > 1.2) contributing to the tree species group separations observed in the PLS-DA model analyses**. Significant differences between the chemical class and subclass abundance in the four analysed trees are presented. p-values were obtained from a one-way ANOVA.

|  | **VIP PLS-DA score** | | **ANOVA** | | |
| --- | --- | --- | --- | --- | --- |
| **Chemical class** | **M9.VIP[3]** | **M9.VIP[3]cvSE * 2.44693** | **p-value** | **F** | **R2** |
| Azoles | 1.46 | 1.05 | <0.0001 | 52105 | 0.9999 |
| Benzene and substituted derivatives | 3.01 | 1.32 | <0.0001 | 1688 | 0.9984 |
| Carboxylic acids and derivatives | 2.69 | 0.91 | <0.0001 | 33.61 | 0.9265 |
| Cinnamic acids and derivatives | 1.95 | 0.53 | <0.0001 | 1047 | 0.9975 |
| Coumarins and derivatives | 4.73 | 2.71 | <0.0001 | 223.1 | 0.9882 |
| Fatty Acyls | 1.53 | 0.77 | <0.0001 | 259.1 | 0.9898 |
| Flavonoids | 4.63 | 3.76 | <0.0001 | 19181 | 0.9999 |
| Furanoid lignans | 1.36 | 0.75 | <0.0001 | 26739 | 0.9999 |
| Isoflavonoids | 1.90 | 1.84 | <0.0001 | 16011 | 0.9998 |
| Lignan glycosides | 1.21 | 0.33 | <0.0001 | 897.1 | 0.997 |
| Prenol lipids | 3.35 | 1.27 | <0.0001 | 227.8 | 0.9884 |
| Pyrans | 2.58 | 1.29 | <0.0001 | 546.5 | 0.9951 |
| Tannins | 1.51 | 1.04 | <0.0001 | 1834 | 0.99 |
| **Chemical subclass** | **M5.VIP[3]** | **M5.VIP[3]cvSE * 2.44693** | **p-value** | **F** | **R2** |
| 1-hydroxy-2-unsubstituted benzenoids | 1.75162 | 0.978371 | <0.0001 | 342.9 | 0.9923 |
| Alcohols and polyols | 2.53317 | 1.4875 | <0.0001 | 787.5 | 0.9966 |
| Amines | 3.17939 | 1.79958 | <0.0001 | 123.2 | 0.9788 |
| Amino acids- peptides- and analogues | 3.26313 | 2.94227 | 0.0005 | 19.43 | 0.8793 |
| Anisoles | 1.27563 | 0.786665 | <0.0001 | 1558 | 0.9983 |
| Benzoic acids and derivatives | 2.1616 | 1.26994 | <0.0001 | 3293 | 0.9992 |
| Biflavonoids and polyflavonoids | 1.81853 | 1.89176 | <0.0001 | 7789 | 0.9997 |
| Carbohydrates and carbohydrate conjugates | 2.92821 | 3.15446 | <0.0001 | 926.1 | 0.9971 |
| Carbonyl compounds | 1.67358 | 0.383037 | <0.0001 | 5268 | 0.9995 |
| Coumarin glycosides | 3.52226 | 3.74477 | <0.0001 | 157.2 | 0.9833 |
| Diphenylmethanes | 1.3736 | 0.847465 | <0.0001 | 1709 | 0.9984 |
| Diterpenoids | 1.46316 | 0.791013 | <0.0001 | 178.4 | 0.9853 |
| Ethers | 1.95471 | 1.7845 | 0.0002 | 27.03 | 0.9102 |
| Fatty acyl glycosides | 1.24231 | 0.79363 | <0.0001 | 1334 | 0.998 |
| Flavans | 2.74916 | 1.96514 | <0.0001 | 18228 | 0.9999 |
| Flavones | 1.15559 | 1.30776 | <0.0001 | 50504 | 0.9999 |
| Flavonoid glycosides | 3.31803 | 3.54941 | <0.0001 | 3543 | 0.9992 |
| Furofuran lignans | 1.45749 | 1.3585 | <0.0001 | 3E+05 | 0.9999 |
| Halobenzenes | 1.49034 | 0.860809 | <0.0001 | 601.1 | 0.9956 |
| Hexacarboxylic acids and derivatives | 1.29164 | 1.47213 | <0.0001 | 1663 | 0.9984 |
| Hydrolysable tannins | 1.7698 | 1.33799 | <0.0001 | 2028 | 0.9987 |
| Hydroxycinnamic acids and derivatives | 2.10689 | 1.15987 | <0.0001 | 974.8 | 0.9973 |
| Hydroxycoumarins | 4.39675 | 3.06436 | <0.0001 | 44.61 | 0.9436 |
| Imidazoles | 1.81267 | 1.00322 | <0.0001 | 65107 | 0.9999 |
| Isoflavonoid O-glycosides | 2.33997 | 2.65465 | <0.0001 | 11033 | 0.9998 |
| Methoxyphenols | 1.4279 | 1.02388 | <0.0001 | 2308 | 0.9988 |
| N-phenylureas | 3.35229 | 2.49236 | <0.0001 | 2385 | 0.9989 |
| O-methylated flavonoids | 3.41454 | 3.44067 | <0.0001 | 5986 | 0.9996 |
| Pentacarboxylic acids and derivatives | 1.81515 | 2.0567 | <0.0001 | 6739 | 0.9996 |
| Pyranones and derivatives | 1.63808 | 1.32719 | <0.0001 | 11062 | 0.9998 |
| Sesquiterpenoids | 1.19802 | 0.389413 | <0.0001 | 6652 | 0.9996 |
| Terpene glycosides | 2.88571 | 2.27152 | <0.0001 | 5384 | 0.9995 |
| Triterpenoids | 3.60937 | 2.58653 | <0.0001 | 183.5 | 0.9857 |

**Table S13. Descriptive data and RSD of protocols 12, 14 and 18 on each tree dataset.** In the table, the value of detected features is the average of features observed on the triplicates of each protocol ± standard deviation. Features detected in all the triplicates were considered “consistent features” and were used to calculate the mean relative standard deviation, RSD. Features with an RSD higher than 30% were excluded from the analysis mentioned above.

| **Dataset** | **Protocol** | **# Detected features** | **# Identified Chemical classes*** | **# of features detected on 3/3 replicates** | **Mean RSD of consistent features** | **# Features with RSD > 30%** |
| --- | --- | --- | --- | --- | --- | --- |
| **Cherry** | 12 | 1813.3 ± 28.9 | 73 | 1750 | 17.2% | 298 |
|  | 18 | 1875 ± 17.05 | 75 | 1817 | 7.03% | 139 |
| **Ash** | 12 | 3702.6 ± 25 | 94 | 3482 | 8.9% | 391 |
|  | 18 | 3724.6 ± 16.8 | 95 | 3545 | 9.5% | 393 |
| **Horse-chestnut** | 12 | 1687 ± 4 | 75 | 1663 | 9.8% | 352 |
|  | 18 | 1714 ± 4.9 | 75 | 1688 | 10% | 319 |
| **Oak** | 14 | 3860.3 ± 47 | 83 | 3639 | 18.1% | 1126 |
|  | 18 | 4062.6 ± 7.5 | 83 | 3952 | 9.4% | 400 |

*The reported number of chemical classes was based on the ClassyFire chemical ontology. The data includes the “Unknown” group (Features that were not classified or whose classification threshold was below 50%).


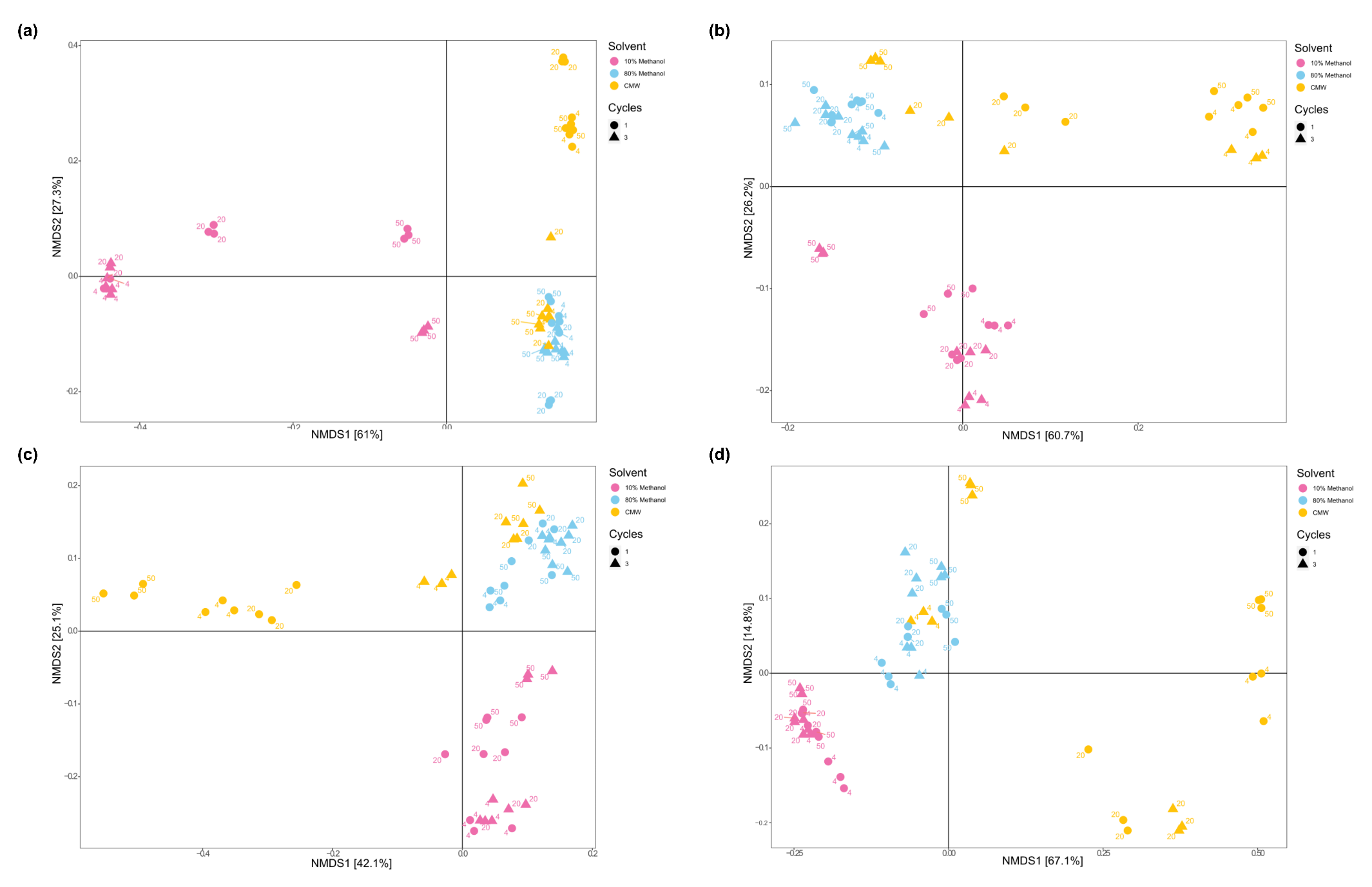


**Fig. S1 Impact of extraction conditions on β-diversity of the crude extracts obtained from (a) ash, (b) cherry, (c) horse chestnut and (d) oak wood (Bray–Curtis index visualized with non-metric multidimensional scaling (NMDS).**


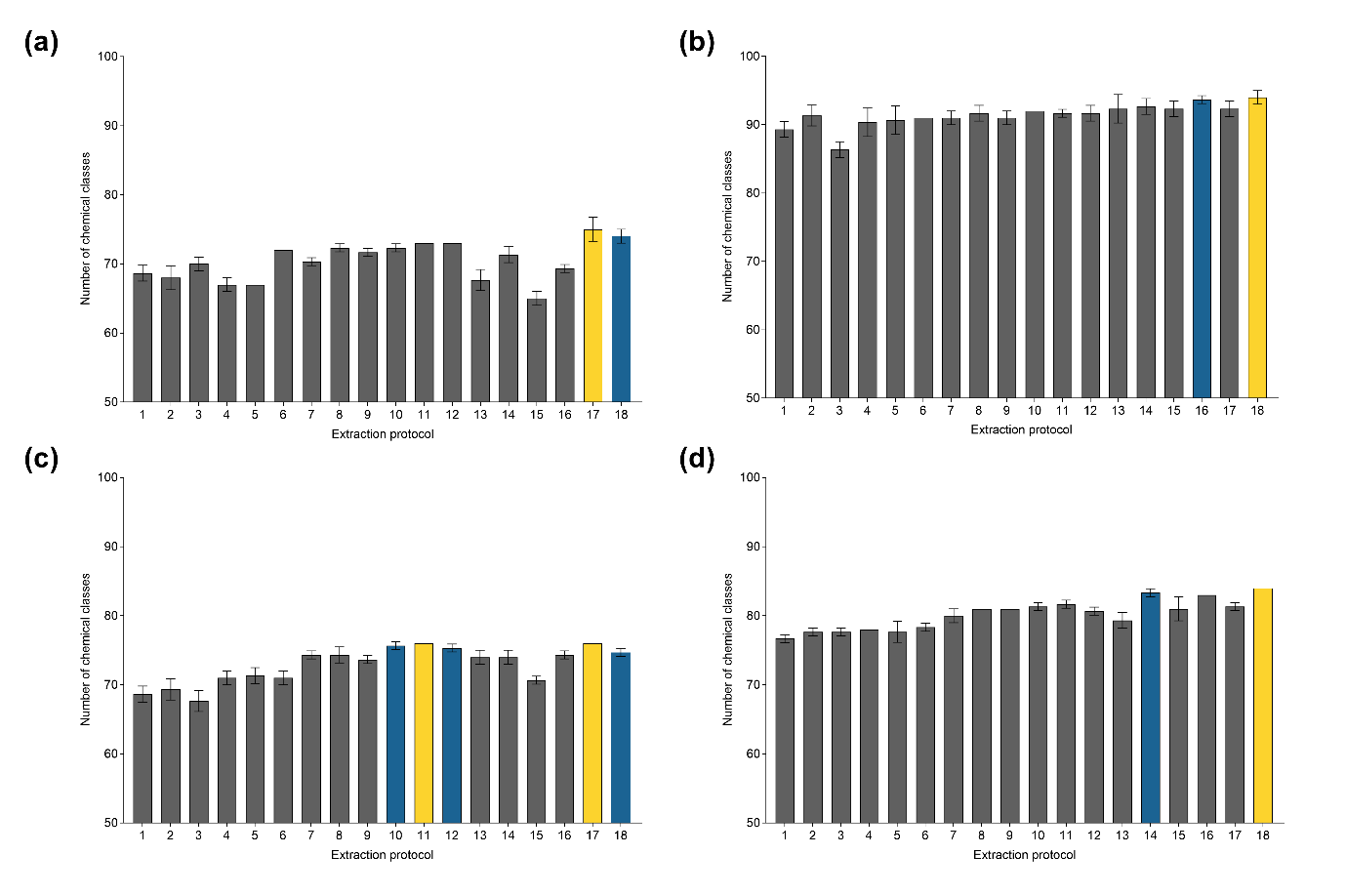


**Fig. S2 Number of chemical classes identified on the evaluated protocols on the (a) cherry, (b) ash, (c) horse chestnut, and (d) oak dataset.** Tree biological replicates’ average and standard deviation (error bars) is presented. Bar-coloured orange or blue represents each dataset’s highest (yellow) and second-highest (blue) values.


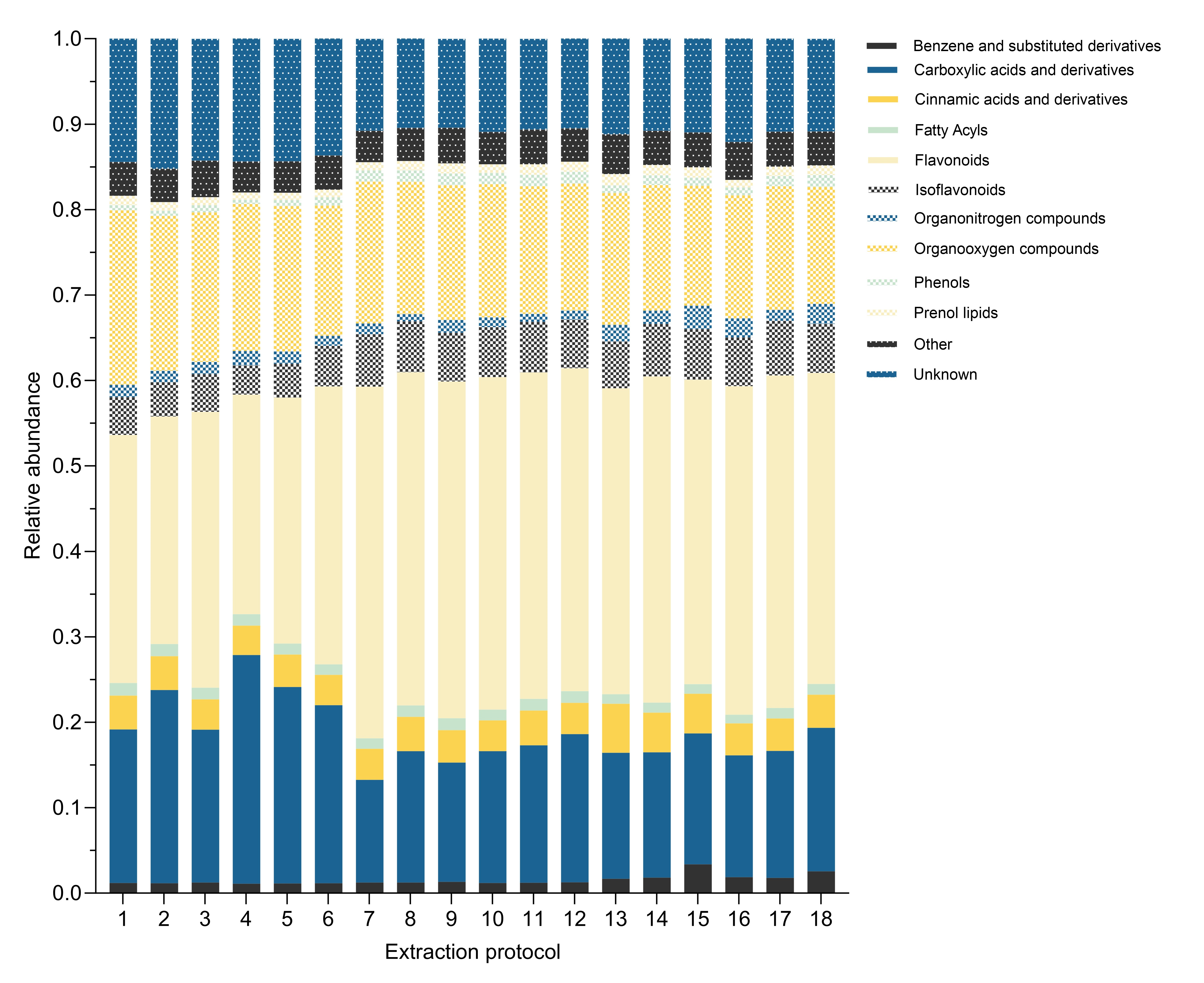


**Fig. S3 Stacked bar plot of chemical class level taxa (ClassyFire) in the cherry dataset.** Stacked bar figure illustrates the relative abundance of identified taxa at a Class level across individual evaluated extraction protocols. In the figure, those chemical classes whose relative abundance was lower than 0.5% were merged as "Other" class groups. Features whose classification score in SIRIUS was lower than 50% (probability < 0.5) and features with no classification were included in the " Unknown " group.


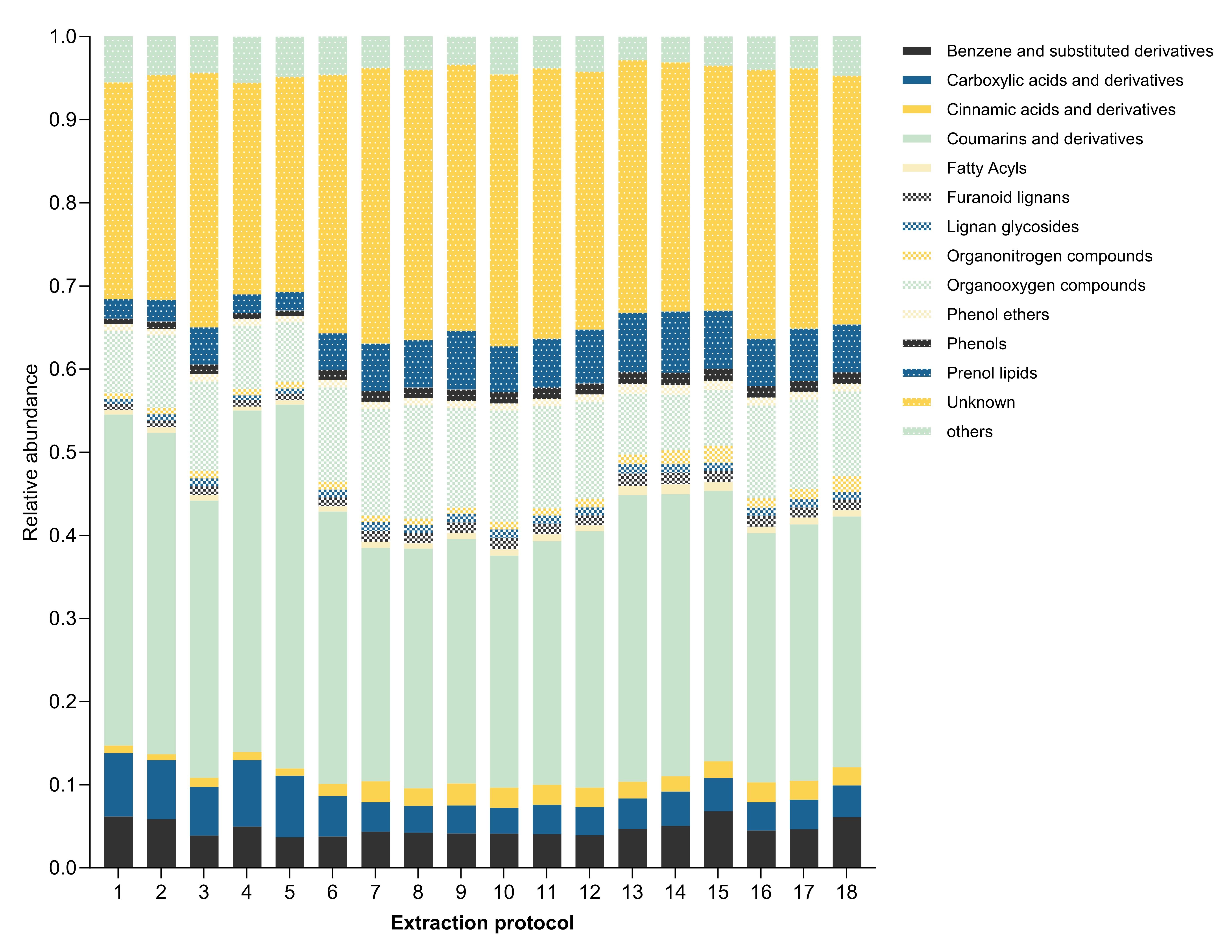


**Fig. S4 Stacked bar plot of chemical class level taxa (ClassyFire) in the ash dataset.** Stacked bar figure illustrates the relative abundance of identified taxa at a Class level across individual evaluated extraction protocols. In the figure, those chemical classes whose relative abundance was lower than 0.5% were merged as "Other" class groups. Features whose classification score in SIRIUS was lower than 50% (probability < 0.5) and features with no classification were included in the " Unknown " group.


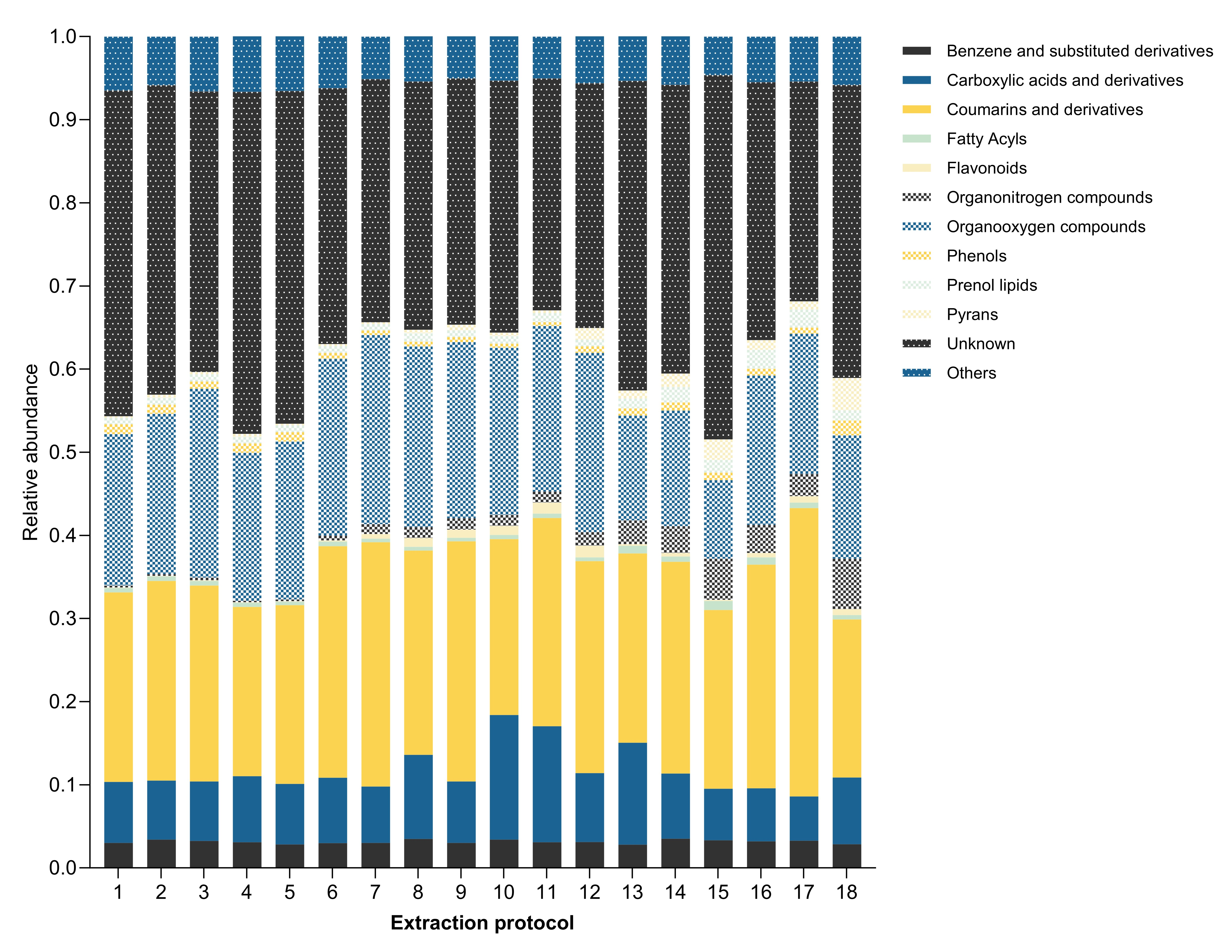


**Fig. S5 Stacked bar plot of chemical class level taxa (ClassyFire) in the horse chestnut dataset.** Stacked bar figure illustrates the relative abundance of identified taxa at a Class level across individual evaluated extraction protocols. In the figure, those chemical classes whose relative abundance was lower than 0.5% were merged as "Other" class groups. Features whose classification score in SIRIUS was lower than 50% (probability < 0.5) and features with no classification were included in the "Unknown" group.


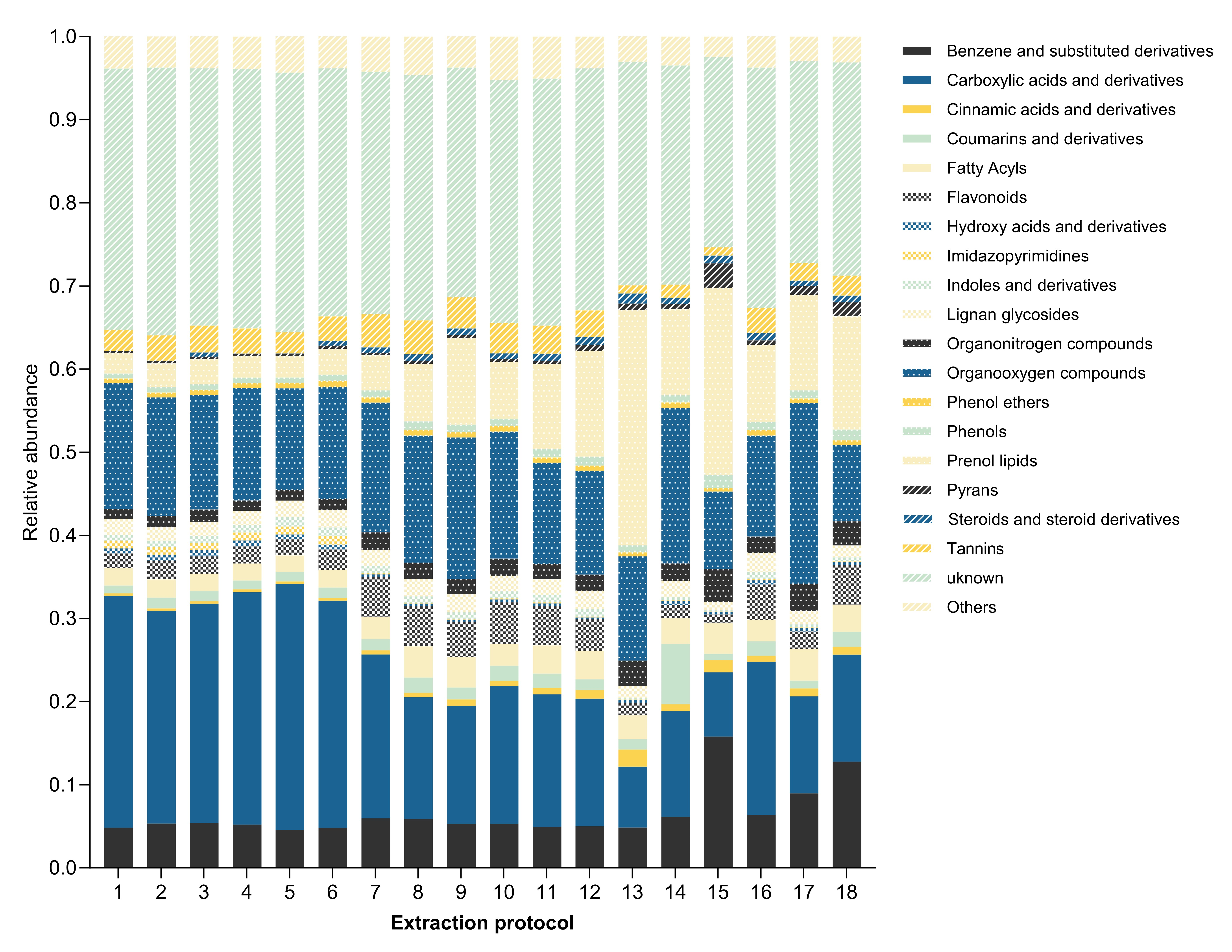


**Fig. S6 Stacked bar plot of chemical class level taxa (ClassyFire) in the oak dataset.** Stacked bar figure illustrates the relative abundance of identified taxa at a Class level across individual evaluated extraction protocols. In the figure, those chemical classes whose relative abundance was lower than 0.5% were merged as "Other" class groups. Features whose classification score in SIRIUS was lower than 50% (probability < 0.5) and features with no classification were included in the " Unknown " group.


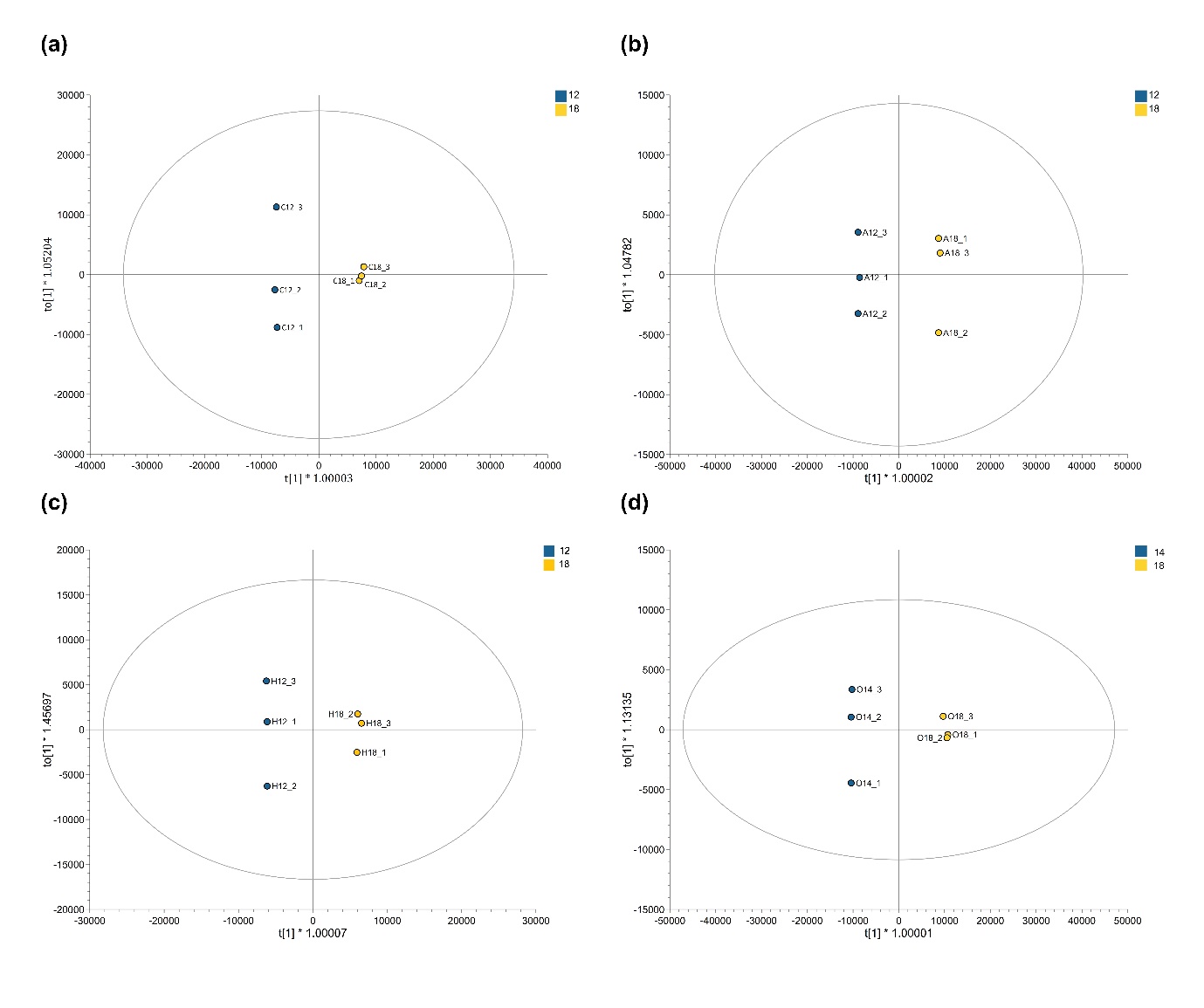


**Fig. S7** **OPLS-DA model score plots for (a) cherry, (b) ash, (c) horse chestnut and (d) oak wood extracts obtained with protocols 12, 14 and 18.** The horizontal axis represents the predicted score of the first component, which explains 55.7%, 71.6%, 60.6%, and 91% of the between-group variations in the cherry, ash, horse chestnut and oak models, respectively. The vertical axis represents the orthogonal principal component score, which explained 35%, 9%, 21.1%, and 4.8% of the within-group variations in the cherry, ash, horse chestnut, and oak models.


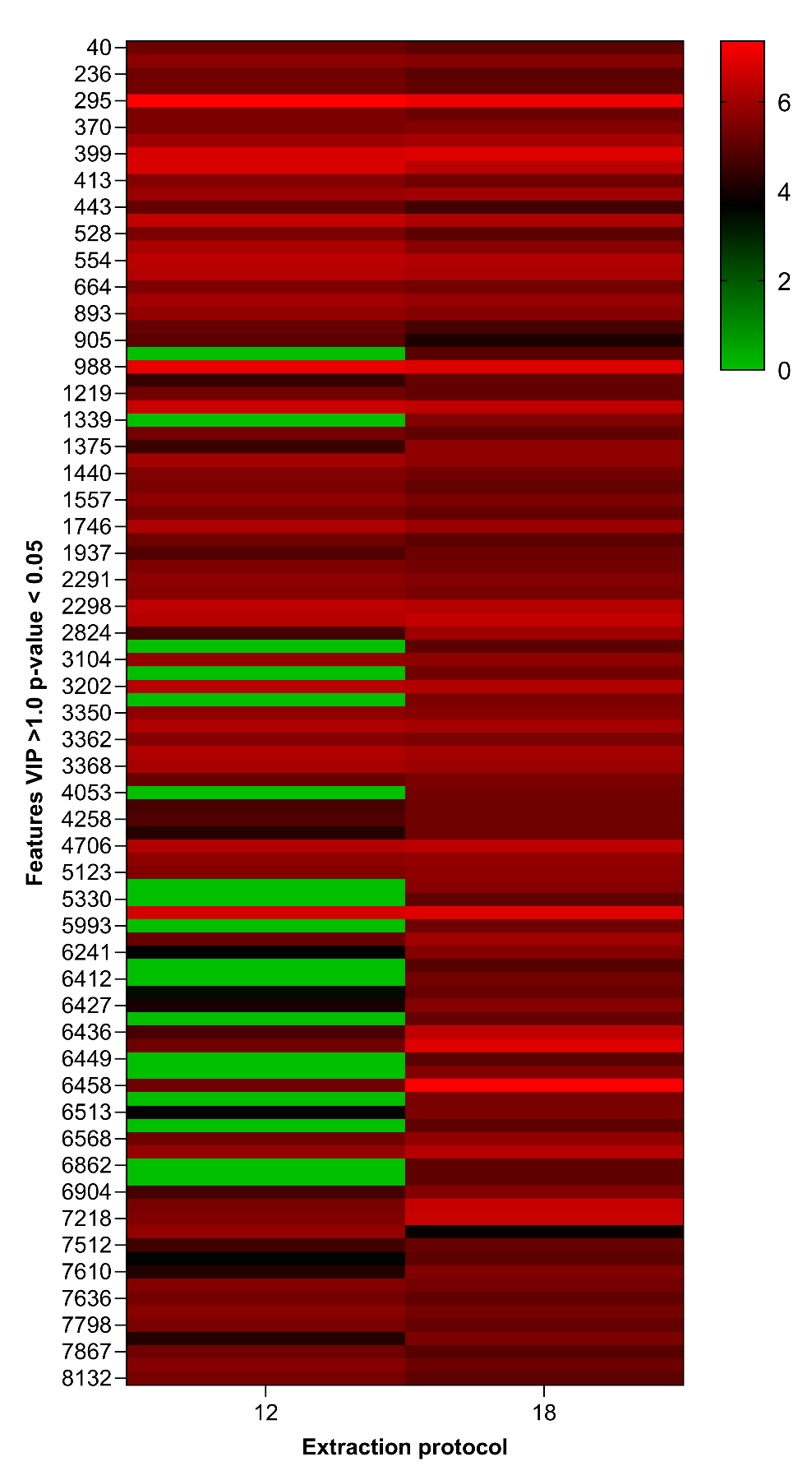


**Fig. S8 Heatmap of the relative abundance of selected features that discriminate between protocols 12 and 18 in the cherry dataset.** The Figure represents the log10 of the peak area (features relative abundance) by the colour scale. Only features with a VIP score higher than 1.0 and a significant difference among protocols are presented (p-values <0.05).


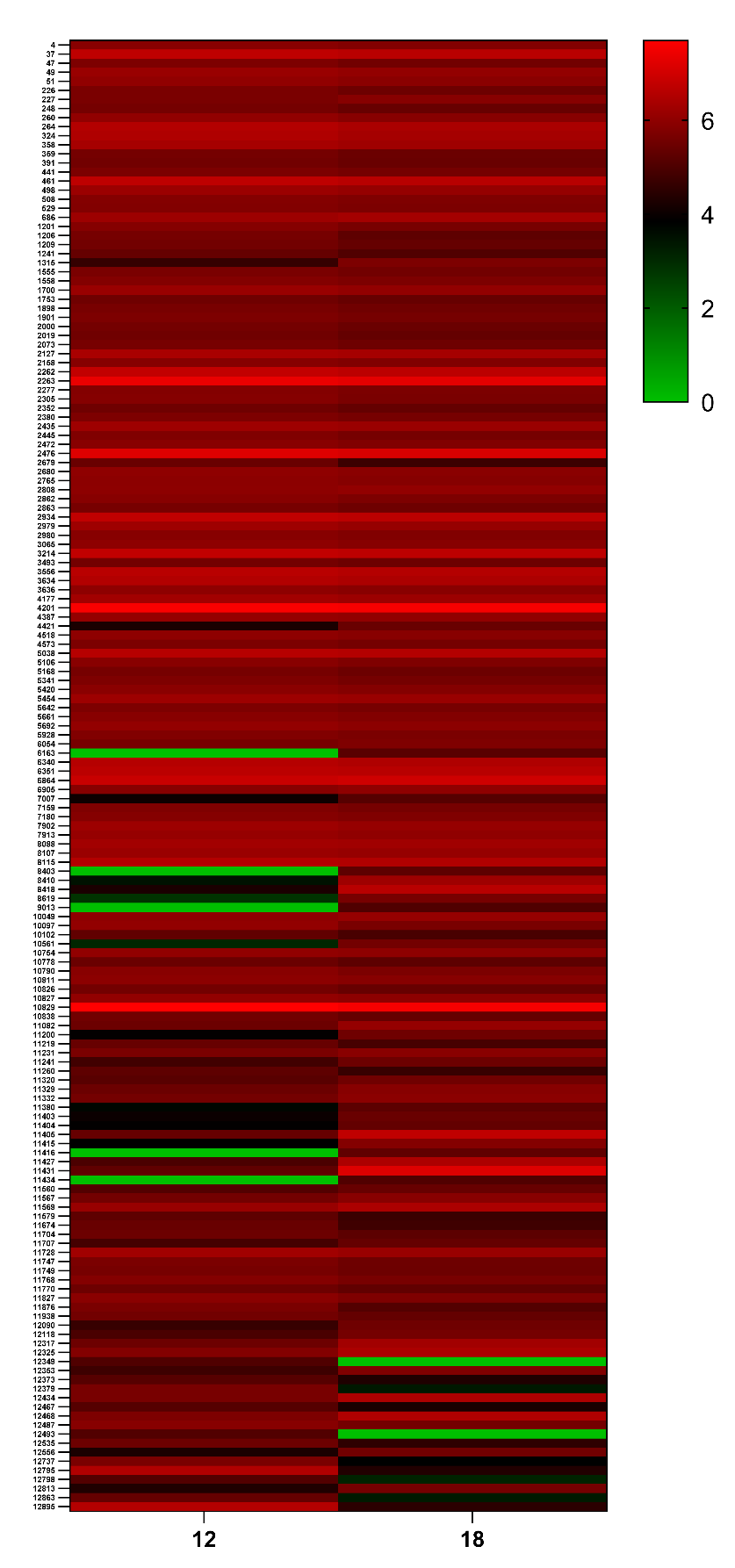


**Fig. S9 Heatmap of the relative abundance of selected features that discriminate between protocols 12 and 18 in the ash dataset.** The Figure represents the log10 of the peak area (features relative abundance) by the colour scale. Only features with a VIP score higher than 1.0 and a significant difference among protocols are presented (p-values <0.05).


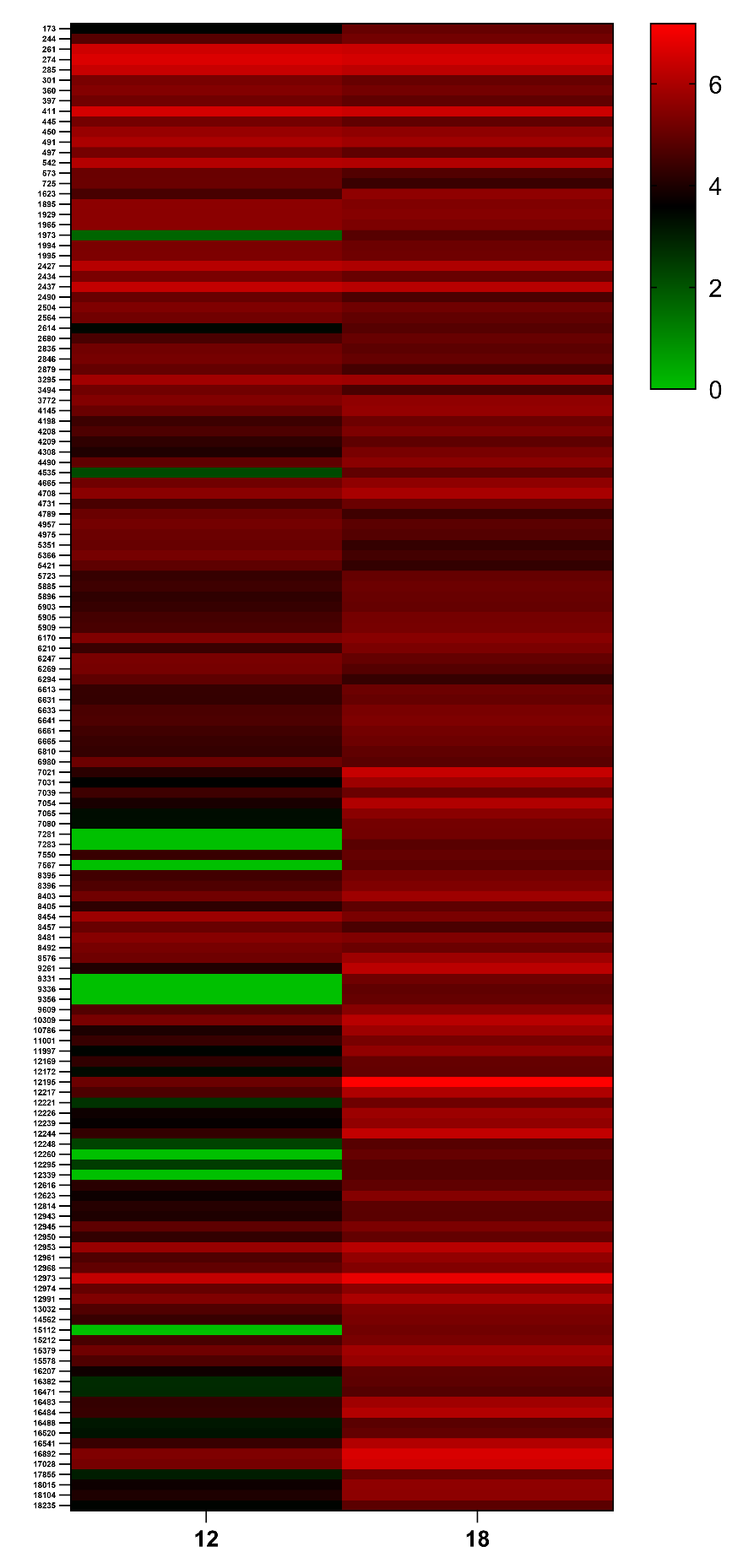


**Fig. S10 Heatmap of the relative abundance of selected features that discriminate between protocols 12 and 18 in the horse chestnut dataset.** The Figure represents the log10 of the peak area (features relative abundance) by the colour scale. Only features with a VIP score higher than 1.0 and a significant difference among protocols are presented (p-values <0.05).


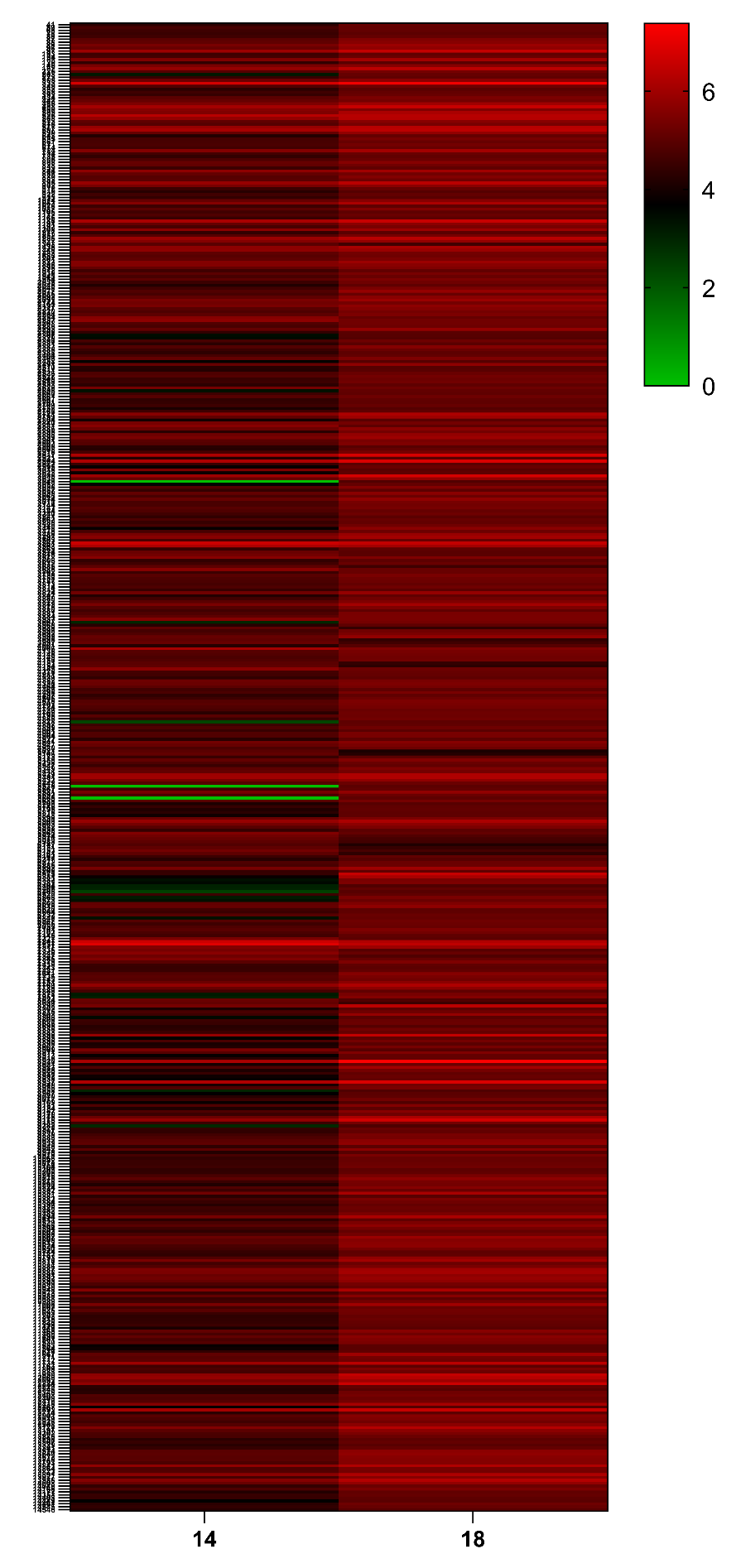


**Fig. S11 Heatmap of the relative abundance of selected features that discriminate between protocols 14 and 18 in the oak dataset.** The Figure represents the log10 of the peak area (features relative abundance) by the colour scale. Only features with a VIP score higher than 1.0 and a significant difference among protocols are presented (p-values <0.05).


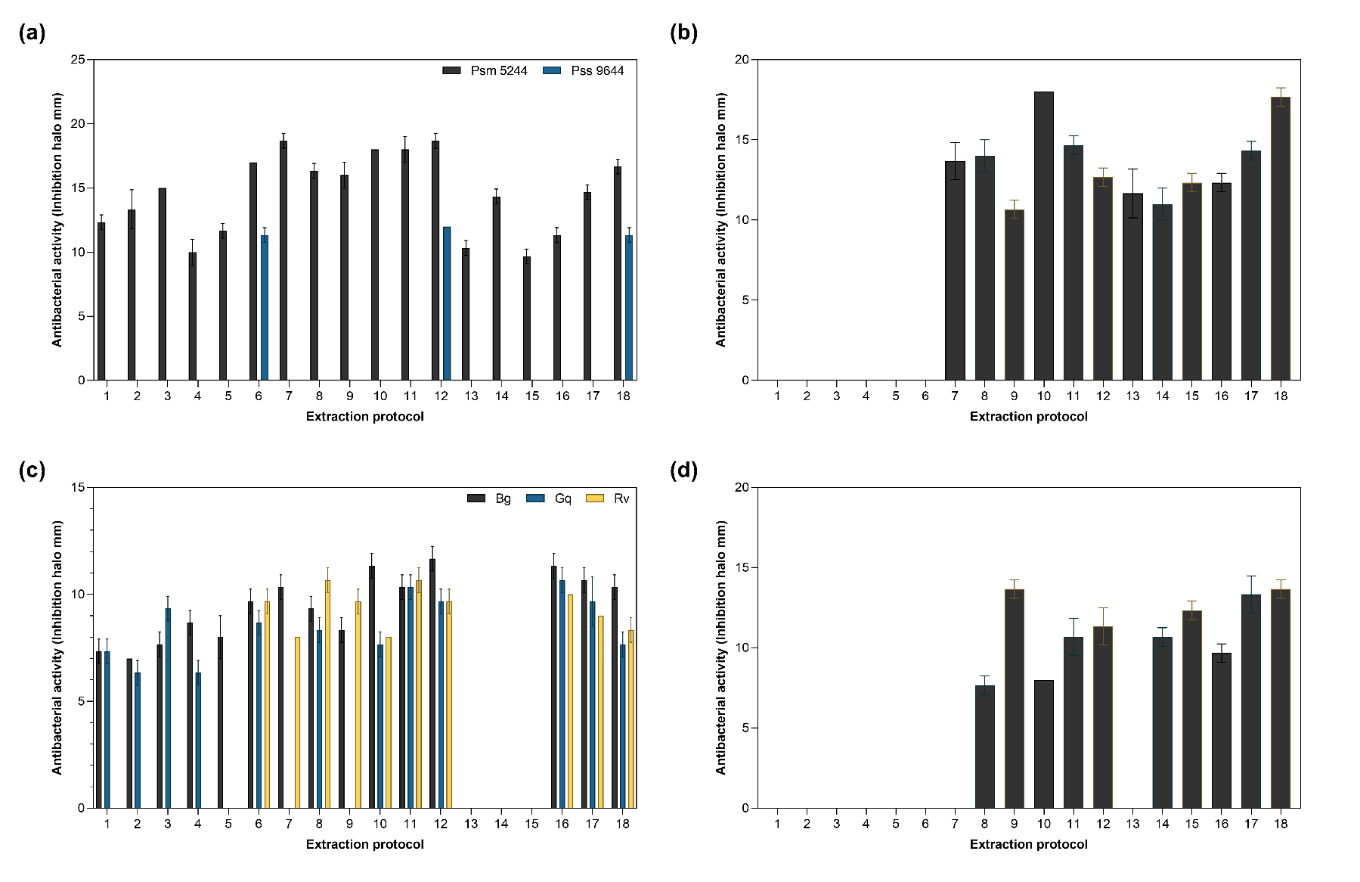


**Fig. S12 Antibacterial activity of wood extracts against their cognate pathogen(s).** (a) Activity of cherry wood extracts against *Pseudomonas syringae* pv. *syringae* (*Pss* 9644) and *P. amygdali* pv. *morsprunorum* (*Psm* 5244); (b) activity of ash wood extracts against *P. savastanoi* pv. *fraxini* (*Psf* 1006); (c) activity of oak wood extracts against *Rahnella victoriana* (*Rv* BRK18a), *Brenneria goodwinii* (*Bg* FRB171), and *Gibbsiella quercinecans* (*Gq* FRB124); (d) ctivity of horse chestnut wood extracts against *P. syringae* pv. *aesculi* (*Pae* 2250). Values represent the average of three biological replicates' average and error bars show the standard deviation.


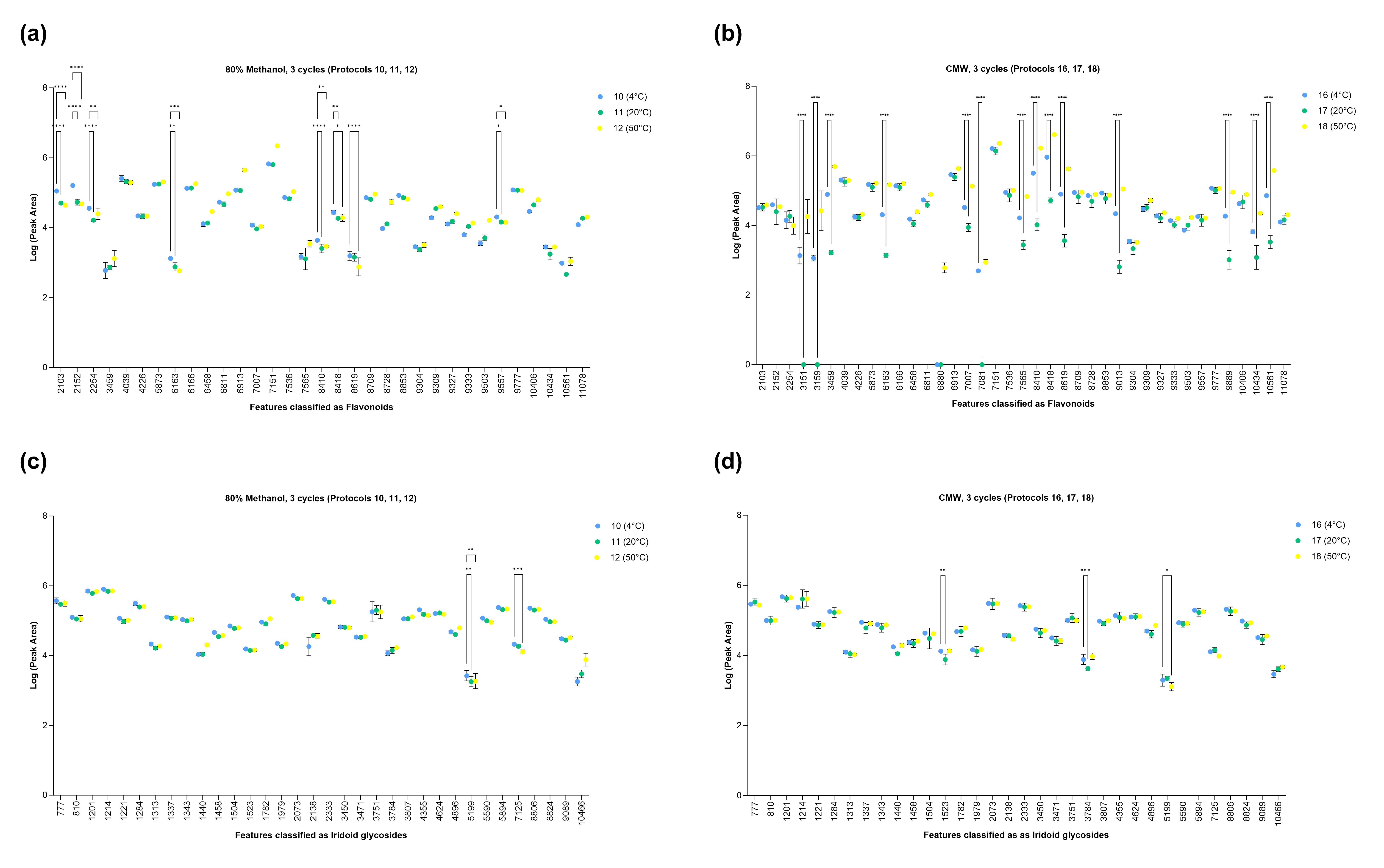


**Fig. S13** **Relative abundance of features classified as flavonoids (a, b) and iridoid glycosides (c, d) in the ash dataset.** A two-way ANOVA, followed by Dunnett’s multiple comparison test, was performed to evaluate the effect of temperature on the feature's abundance. Protocols that used three extraction cycles and methanol (a, c) or CMW (b, d) were chosen for comparison. The average log-transformed peak area from three biological replicates (dots) and standard deviation (error bars) are presented. Features with significantly lower abundance at higher temperatures are shown, indicating the p-value as follows: (*) p≤0.05, (**) p ≤ 0.01, (***) p ≤0.001, (****) p<0.0001.


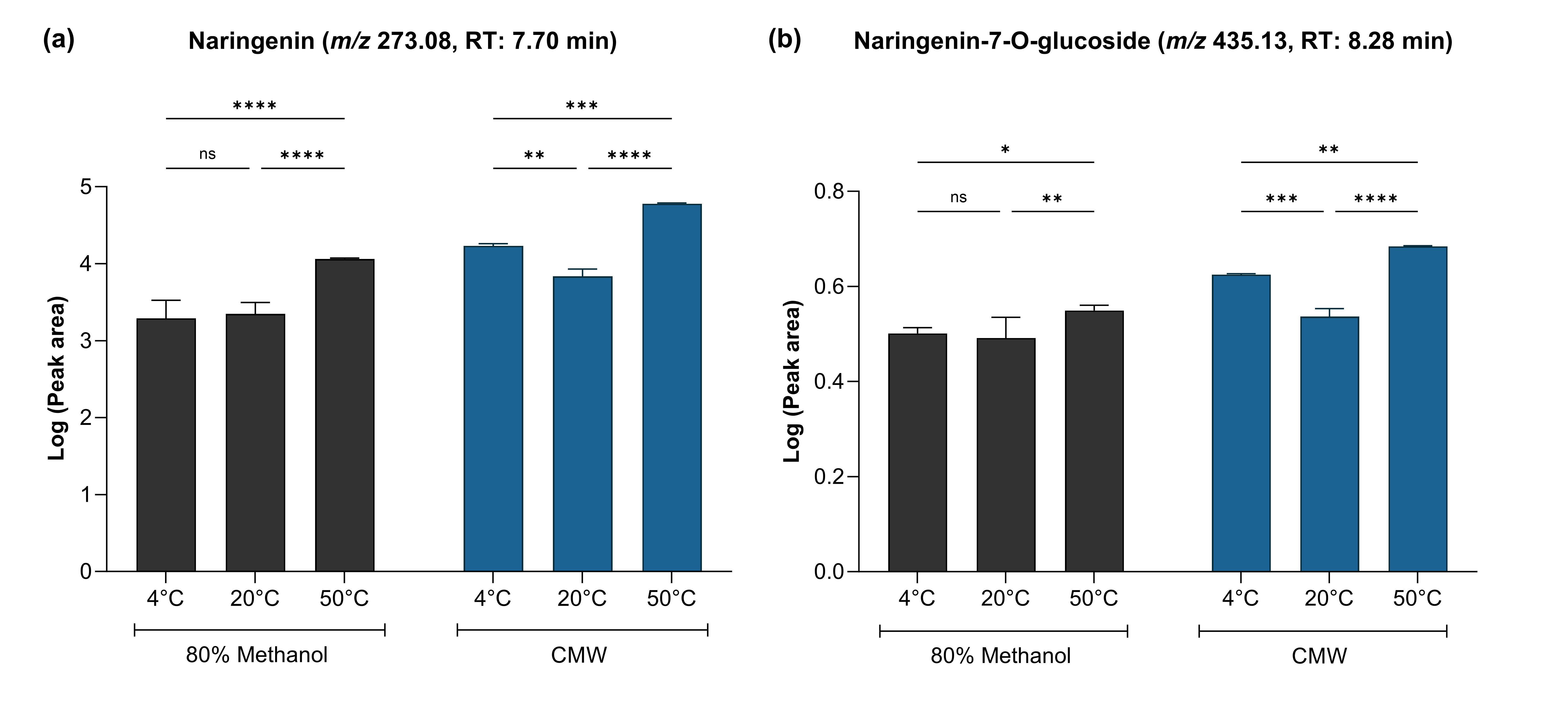


**Fig. S14. Relative abundance of (a) naringenin and (b) naringenin 7-*O*-glycoside obtained by the extraction of ash wood with 80% methanol or CMW at different temperatures during three extraction cycles.** A two-way ANOVA, followed by Dunnett’s multiple comparison test, was performed to evaluate the effect of temperature on the feature's abundance. Protocols that used three extraction cycles and methanol (a) or CMW (b) were chosen for comparison. The average log-transformed peak area from three biological replicates (dots) and standard deviation (error bars) are presented. Statistical differences among extraction protocols are indicated by the *p*-value as follows: (ns) no significant p>0.05, (*) p≤0.05, (**) p ≤ 0.01, (***) p ≤0.001, (****) p<0.0001. The metabolites annotation was performed on SIRIUS 4.0 based on the features’ MSMS fragmentation profile. Metabolite annotation is considered as level 3 as defined by the Metabolomics Standards Initiative.


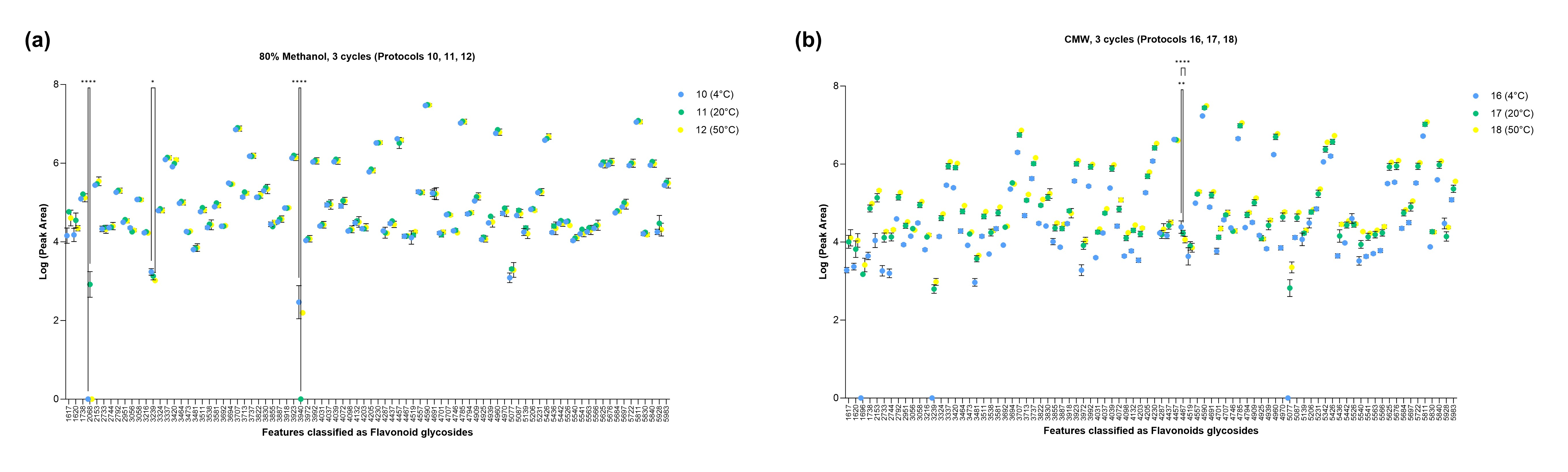


**Fig. S15 Relative abundance of features classified as flavonoid glycosides in the cherry dataset.** A two-way ANOVA, followed by Dunnett’s multiple comparison test, was performed to evaluate the effect of temperature on the feature's abundance. Protocols that used three extraction cycles and methanol (a) or CMW (b) were chosen for comparison. The average log-transformed peak area from three biological replicates (dots) and standard deviation (error bars) are presented. Features with significantly lower abundance at higher temperatures are shown, indicating the *p*-value as follows: (*) p≤0.05, (**) p ≤ 0.01, (***) p ≤0.001, (****) p<0.0001


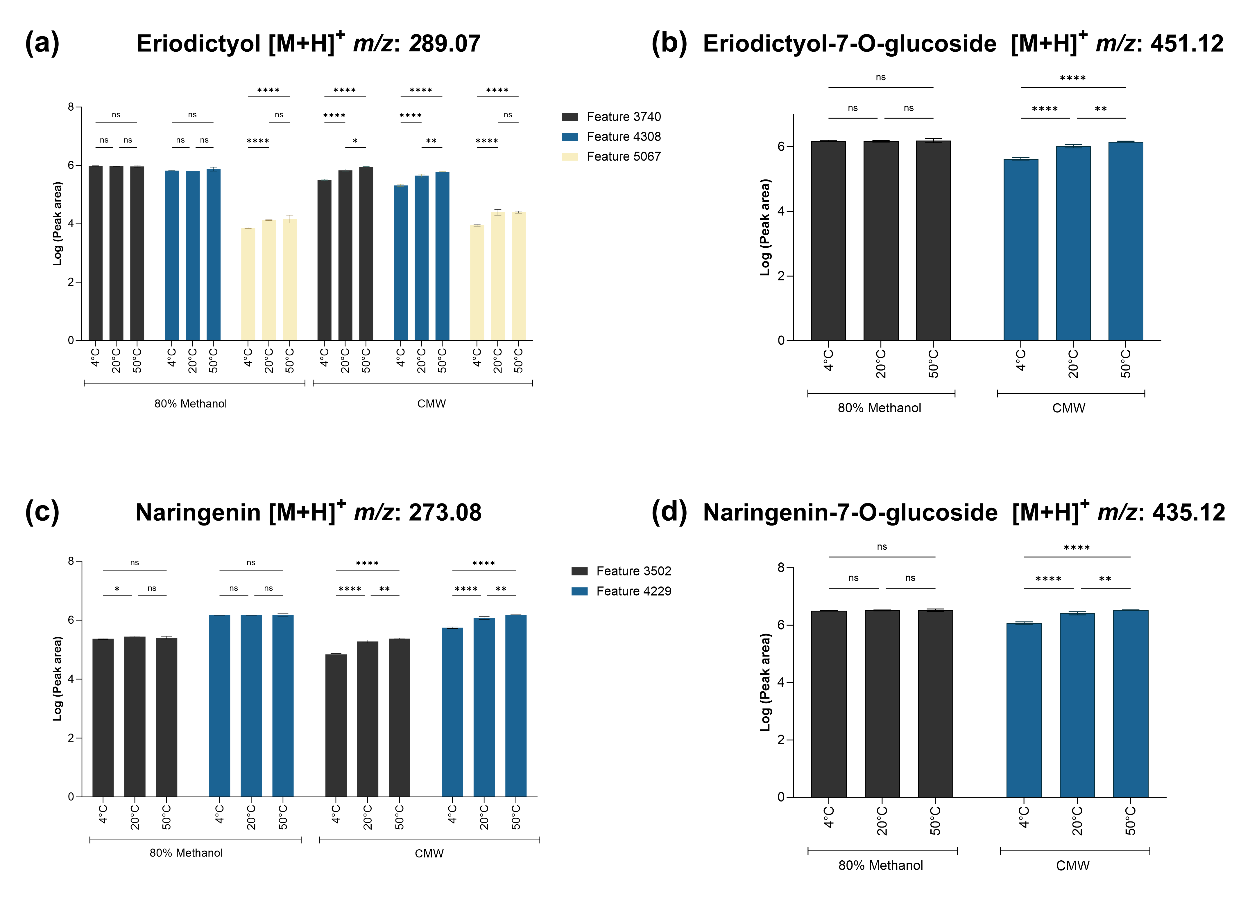


**Fig. S16 Relative abundance of (a) eriodictyol, (b) eriodictyol 7-*O*-glucoside, (c) naringenin and (d) naringenin 7-*O*-glucoside obtained by the extraction of cherry wood with 80% methanol or CMW at different temperatures during three extraction cycles.** A two-way ANOVA, followed by Dunnett’s multiple comparison test, was performed to evaluate the effect of temperature on the feature's abundance. Protocols that used three extraction cycles and methanol or CMW were chosen for comparison. The average log-transformed peak area from three biological replicates and standard deviation (error bars) are presented. Statistical differences among extraction protocols are indicated by the *p*-value as follows: (ns) no significant p>0.05, (*) p≤0.05, (**) p ≤ 0.01, (***) p ≤0.001, (****) p<0.0001. The metabolites annotation was performed on SIRIUS 4.0 based on the features’ MSMS fragmentation profile. Metabolite annotation is considered to be level 3 as defined by the Metabolomics Standards Initiative.
